# Molecular and genetic evidence for the role of AMBRA1 in suppressing S-phase entry and tumorigenesis

**DOI:** 10.1016/j.isci.2025.113054

**Published:** 2025-07-05

**Authors:** Hisako Akatsuka, Tomohiro Kashikawa, Kaori Masuhara, Mizuki Tokusanai, Chenyang Li, Yumi Iida, Chisa Okada-Yamaguchi, Yoshinori Okada, Masayuki Tanaka, Takahiro Suzuki, Norio Yamamoto, Katsuto Hozumi, Tomoaki Tanaka, Hirofumi Nakaoka, Kazuyoshi Hosomichi, Yu Hamaguchi, Michiaki Hamada, Yoshiki Shiraishi, Akihide Kamiya, Yoshihiko Nakamura, Kaito Harada, Abd Aziz Ibrahim, Takashi Yahata, Masato Ohtsuka, Naoya Nakamura, Hiroyuki Hosokawa, Minoru Kimura, Ituro Inoue, Takehito Sato

**Affiliations:** 1 Division of Host Defense Mechanism, Tokai University School of Medicine, Isehara, Kanagawa 259-1193, Japan; 2 Medical Science College Office, Tokai University, Isehara, Kanagawa 259-1193, Japan; 3 Department of Ophthalmology, Tokai University School of Medicine, Isehara, Kanagawa 259-1193, Japan; 4 Department of Hematology and Oncology, Tokai University School of Medicine, Isehara, Kanagawa 259-1193, Japan; 5 Department of Molecular Life Science, Tokai University School of Medicine, Isehara, Kanagawa 259-1193, Japan; 6 Department of Pathology, Tokai University School of Medicine, Isehara, Kanagawa 259-1193, Japan; 7 Translational Molecular Therapeutics Laboratory, Division of Host Defense Mechanism, Tokai University School of Medicine, Isehara, Kanagawa 259-1193, Japan; 8 Institute of Medical Sciences, Tokai University School of Medicine, Isehara, Kanagawa 259-1193, Japan; 9 Division of Pulmonary Medicine, Department of Medicine, Tokai University School of Medicine, Isehara, Kanagawa 259-1193, Japan; 10 Department of Molecular Diagnosis, Graduate School of Medicine, Chiba University, Chuo-ku, Chiba 260-8670, Japan; 11 Human Genetics Laboratory, National Institute of Genetics, Mishima, Shizuoka 411-8540, Japan; 12 Department of Cancer Genome Research, Sasaki Institute, Chiyoda-ku, Tokyo 101-0062, Japan; 13 Department of Bioinformatics & Genomics, Kanazawa University, Kanazawa, Ishikawa 920-8640, Japan; 14 Faculty of Science and Engineering, Waseda University, 55N-06-10, 3-4-1 Okubo Shinjuku-ku, Tokyo 169-8555, Japan; 15 AIST-Waseda University Computational Bio Big-Data Open Innovation Laboratory (CBBD-OIL), 3-4-1, Okubo Shinjuku-ku, Tokyo 169-8555, Japan; 16 Institute for Medical- Oriented Structural Biology, Waseda University, 2-2, Wakamatsu-cho Shinjuku-ku, Tokyo 162-8480, Japan; 17 Laboratory of Computational Genomics, School of Life Science, Tokyo University of Pharmacy and Life Sciences, 1432-1 Horinouchi, Hachioji, Tokyo 192-0392, Japan; 18 Faculty of Nursing, Soka University 1-236, Tangi-machi, Hachioji, Tokyo 192-8577, Japan

**Keywords:** Cell biology, Cancer

## Abstract

AMBRA1, which was initially reported to be essential for nervous system development via autophagy and cell proliferation control, also functions as a tumor suppressor by regulating the ubiquitination of D-type cyclins through interaction with DDB1-Cullin4A/4 B E3 ligase. We had identified a missense mutation in *AMBRA1* through exome analysis of a family with Cowden syndrome. The patient-type mutant showed reduced DDB1 binding and impaired cyclin D degradation. To investigate the physiological role of AMBRA1, we generated *Ambra1* flox mice crossed with Rosa-Cre-ERT2-Tg mice. These inducible *Ambra1* conditional knock out mice exhibited increased body weight, organ size, and enhanced S phase entry, with elevated cyclin D expression in a cell lineage- or differentiation-specific manner. Notably, their susceptibility to spontaneous, radiation-, and chemically induced malignancies was significantly higher. These findings support the role of AMBRA1 as a tumor suppressor that regulates cyclin Ds, although other targets may also contribute.

## Highlights

• *AMBRA1* mutation is identified in no *PTEN* mutated patients with Cowden syndrome
• *Ambra1* cKO mice showed increased body weight and organ size
• Various cells of *Ambra1* cKO mice showed hi-expression of cyclin D and enhanced S-phase entry
• AMBRA1 suppresses tumorigenesis and tumor growth *in vivo* and *in vitro*

## Graphical abstract

**Figure:**
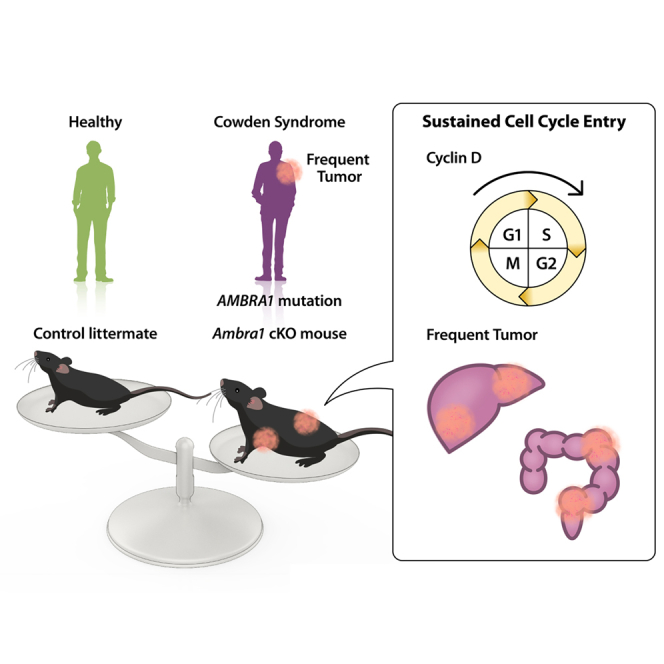

## Introduction

Because multicellular organisms are formed through multiple rounds of cell division, transient or permanent cell proliferation arrest is essential for their integrity.^1^ Improper regulation of the proliferation-quiescence decision affects development and differentiation and leads to disorders such as benign and malignant tumors. Cowden syndrome is a hereditary disorder that was first described in 1963 and exhibits multiple hamartomas or benign tumors in any organ with an increased risk of malignancy.^2^^,^^3^^,^^4^ While mutations in *PTEN* were identified as the cause of Cowden syndrome,^5^ we have recently identified missense mutations in *AMBRA1* by exome analysis of the family of a Cowden syndrome patient without a *PTEN* mutation.^6^

Activating Molecule in Beclin-1 Regulated Autophagy 1 (AMBRA1, also known as DCAF3) was originally reported to be essential for central nervous system development, probably through the control of autophagy and cell proliferation.^7^^,^^8^^,^^9^^,^^10^^,^^11^ We have previously reported that AMBRA1 is involved in cell proliferation arrest independent of autophagy control.^12^ Notably, several different groups have recently reported that AMBRA1 is a master regulator of D-type cyclins and serves as an E3 ubiquitin ligase and a component of the Cullin-RING E3 ligase 4 (CRL4) complex.^13^^,^^14^^,^^15^ Binding of D-type cyclins determines CDK4/6 activity, which determines whether cells enter the proliferative (S) or quiescent (G0/G1) phase.^16^^,^^17^^,^^18^ It has been suggested that AMBRA1 contributes to the proliferation-quiescence decision and tumor suppression.

We first confirmed that the Cowden syndrome patient-type AMBRA1 mutant attenuated the function of controlling the stability of cyclin D and cell proliferation arrest. Although *in vivo* significance of AMBRA1 has not yet been fully explored, in this study, we generated conditional knockout (cKO) mice in which *Ambra1* was systemically disrupted by tamoxifen (TAM) treatment,^19^^,^^20^ and obtained molecular genetic evidence that AMBRA1 is essential for the proliferation-quiescence decision and suppression of tumorigenesis.

## Results

### AMBRA1 mutation found in patients with Cowden syndrome showed impaired capability to control the cell cycle with enhanced stability of cyclin D

**Figure 1:**
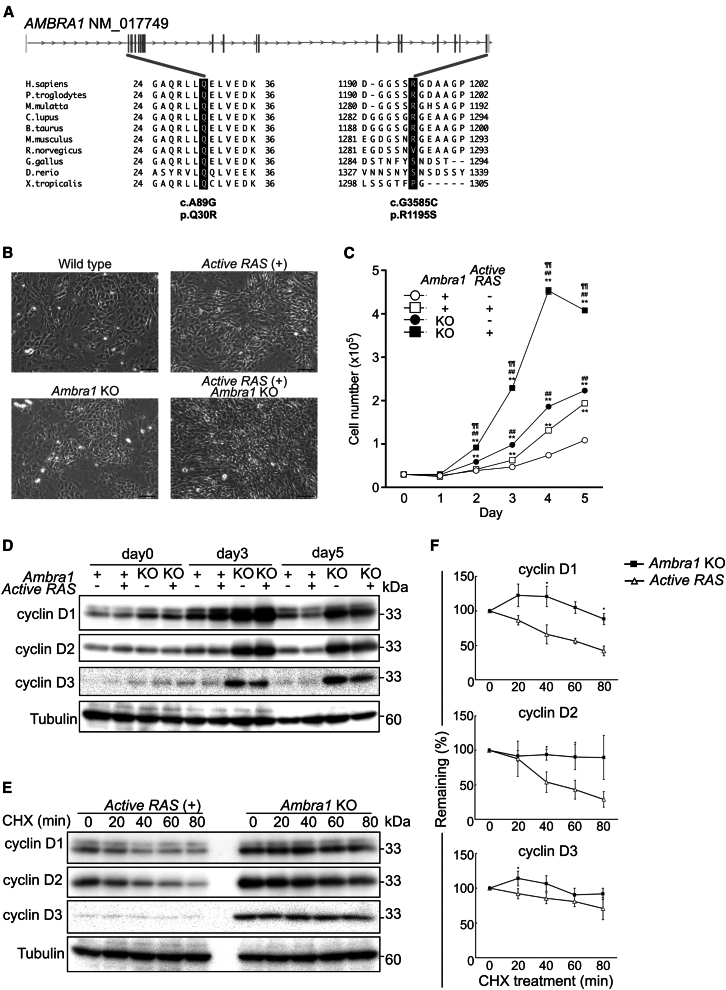
AMBRA1 is involved in controlling the cell cycle and the stability of cyclin Ds (A) AMBRA1 with non-synonymous alterations of A89G:Q30R and G3585C:R1195S appeared exclusively in the symptomatic family members. Sequence alignment of AMBRA1 of Homo sapiens, Pan troglodytes, Macaca mulatta, Canis lupus, Bos taurus, Mus musculus, Rattus norvegicus, Gallus gallus, Danio rerio, and Xenopus tropicalis at the N- and C-terminus. Substitutions found in the patient are indicated. (B) Five days after the culture of MEFs with the indicated genotype with 4-OHT, the cells were observed with a phase contrast inverted microscope (Axio Vert A1, Carl Zeiss). While active RAS-expressing MEFs showed an extended shape, presumably because of the effects on cytoskeletal organization, AMBRA1-deficient cells did not show such a morphological change. Scale bars, 100 μm. (C) The growth curves of MEFs of the indicated genotype after adding 4-OHT at day 0. The values are presented as mean ± standard deviations (SD); *n* = 3 in each group (∗∗*p* < 0.01 compared with the *Ambra1*+ active RAS- group, ^##^*p* < 0.01 compared with the *Ambra1*+ *active RAS*+ group, ^¶¶^*p* < 0.01 compared with the *Ambra1* KO active RAS- group, analyzed by two-way repeated measures ANOVA, followed by Tukey’s post hoc test). Three independent experiments showed similar results. (D) Time-course analysis of cyclin D expression in MEFs of the indicated genotype cultured with 4-OHT. (E) Three days after culture with 4-OHT, MEFs with active RAS expression or *Ambra1* deficiency were cultured with cycloheximide (CHX) for the indicated periods, and extracted protein samples were subjected to immunoblot analysis for cyclin D1, D2, D3, and tubulin. (F) Densitometric analysis of the data in (E) is shown. Closed squares are *Ambra1* KO MEFs, and open triangles are active RAS-expressing MEFs. The expression of cyclin D1, D2, and D3 was higher and more stable in *Ambra1* KO MEFs. Three independent experiments showed similar results. The values are presented as mean ± SD (∗*p* < 0.05 in two-way repeated measures ANOVA, followed by Tukey’s post hoc test).

We had identified a missense mutation in *AMBRA1* by exome analysis of the family of the patient with Cowden syndrome who had multiple neoplasms without Phosphatase and tensin homolog (*PTEN*) mutations.^6^ The patient had two substitutions in AMBRA1 (A89G:Q30R and G3585C:R1195S), and the corresponding amino acids were well-conserved among vertebrate species (Figure 1A). The mutation we identified was not found among the tumor-associated mutations reported in the cBioPortal database, thus suggesting that mutant (A89G:Q30R and G3585C:R1195S) is a novel mutation (Table S1). To explore its physiological significance, we generated *Ambra1* complete KO mice by disrupting exon 2 and found that the mice were embryonically lethal (Figure S1), consistent with previously reported *Ambra1*^*gt/gt*^ mice, which harbor homozygous gene-trapped alleles for *Ambra1*.^7^ Then, we generated *Ambra1*^*flox/flox*^ mice using the “Easi-CRISPR” method.^19^^,^^21^ We inserted two *loxP* sites flanking the intron regions surrounding exon 4 of *Ambra1* and crossed them with Rosa-Cre-ERT2 Tg mice (*Ambra1* cKO mice) to achieve the conditional depletion of *Ambra1* in the presence of TAM or 4-hydroxytamoxifen (4-OHT).

First, to examine the effect of AMBRA1 deficiency on cell growth, we performed *in vitro* experiments using mouse embryonic fibroblasts (MEFs) depleted of AMBRA1, with or without active RAS (H-RAS G12V) expression (Figures 1B–1D). MEFs expressing the active RAS contain a spacer with a stop sequence and a poly(A) signal inserted between exons 1 and 2 flanked by *loxP* sites. Under normal conditions, active RAS expression was suppressed. However, in the presence of 4-OHT, the spacer was excised, allowing RAS expression (Figure S2A). 4-OHT was added to the culture medium to induce the deletion of *Ambra1* exon 4 and expression of active RAS (Figures S2B and S2C). Immunoblot analysis showed that Rb phosphorylation (S807/S811) was increased in AMBRA1 deficient cells while AKT phosphorylation was increased in active RAS expressing cells (Figure S2D). While active RAS-expressing MEFs showed an extended cell shape, presumably due to the effect on cytoskeletal organization, AMBRA1-deficient cells did not show such morphological changes (Figure 1B). However, AMBRA1 deficiency promoted cell proliferation to the same extent as oncogenic H-RAS expression compared to normal MEFs (Figure 1C, closed circles, open squares vs. open circles). Furthermore, AMBRA1 deficiency markedly facilitated the growth of cells expressing active RAS, suggesting a synergistic effect of AMBRA1 depletion and RAS overactivation in promoting cell proliferation (Figure 1C, closed squares).

Recently, several groups have reported that AMBRA1 acts as a master regulator of D-type cyclins and serves as an E3 ubiquitin ligase as a component of the Cullin-RING E3 ligase 4 (CRL4) complexes, resulting in the subsequent proteasomal degradation of cyclin Ds.^13^^,^^14^^,^^15^ As expected, AMBRA1-deficient MEFs expressed higher levels of D-type cyclins than control cells expressing AMBRA1 with or without active RAS expression (Figure 1D). Enhanced expression of D-type cyclins resulting from AMBRA1 depletion was abolished by the addition of exogenous AMBRA1 (Figure S3). To examine the stability of cyclin D proteins, translation was blocked by adding cycloheximide (CHX) to the culture. When *de novo* synthesis was prevented, the expression levels of cyclin D1, D2, and D3 rapidly decreased in active RAS-expressing cells, but were more stable in *Ambra1* KO cells (Figures 1E and 1F).

**Figure 2:**
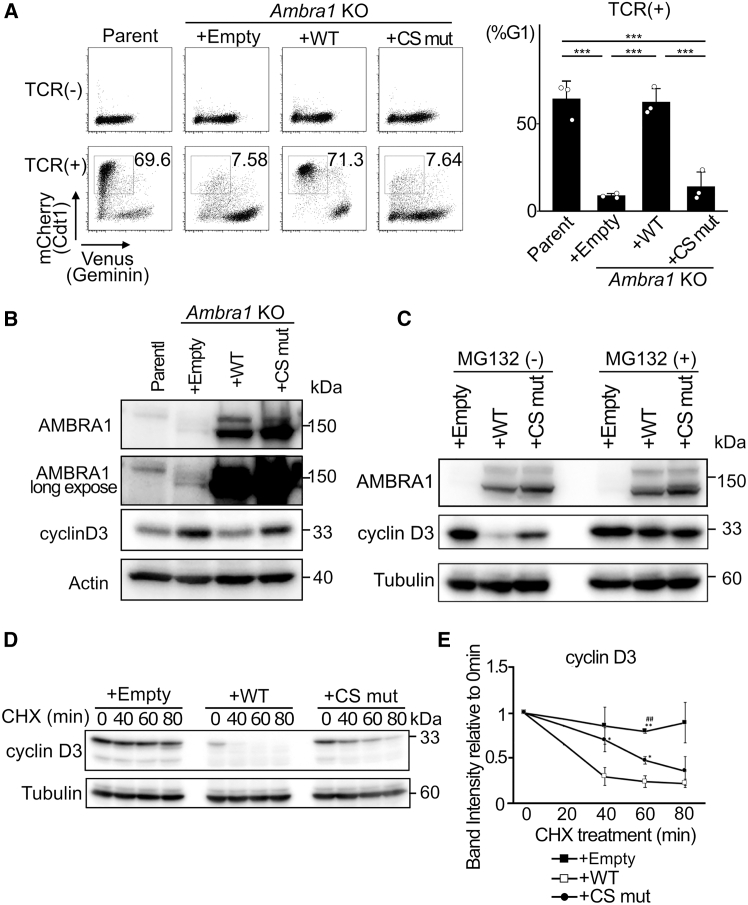
Attenuated function for the control of the cell cycle and cyclin D expression in Cowden syndrome (A) Cell cycle analysis of murine CD4^+^CD8^+^ immature cell line OVA53 (Parent) and *Ambra1*-disrupted OVA53 cells transfected with either empty vector (+Empty), *AMBRA1* wild-type (+WT), or patient-type *AMBRA1* mutant (+CS mut), before (TCR-) and 48 h after TCR/CD3 crosslinking (TCR+). Using the Fucci (SA) system, cells at the G1 phase were visualized to identify the mCherry-positive cells. The percentages of the G1 phase cells are indicated and shown in bar graphs. Means ± SD were calculated using three independent clones per group (∗∗∗*p* < 0.005 in one-way ANOVA followed by Tukey’s post hoc test). (B) Immunoblot analysis of OVA53 cells (Parent), *Ambra1* KO OVA53 cells expressing empty vector (+Empty), AMBRA1 WT (+WT), and patient-type AMBRA1 mutant (+CS Mut). Immunoblotting was performed using antibodies against cyclin D3, AMBRA1, and Actin. (C) Cells used in (B) were cultured in the absence (left) and presence (right) of proteasome inhibitor MG132 at 10 μmol/L for 6 h, lysed, and subjected to immunoblot analysis using antibodies against AMBRA1, cyclin D3, and Tubulin. (D) Following treatment with 25 μg/mL of cycloheximide (CHX) for the indicated time, cell lysates were prepared and subjected to immunoblot analysis using antibodies against cyclin D3 and Tubulin. (E) The relative expressions to the level at 0 min were measured using the *ImageJ* software and represented as mean ± SD; number of samples: *n* = 3. The *p* values were calculated by two-way repeated measures ANOVA, followed by Tukey’s post hoc test. ∗*p* < 0.05 and ∗∗*p* < 0.01 compared with the WT group; #*p* < 0.05 and ##*p* < 0.01 compared with the CS mut group.

We previously reported that *Ambra1*-deficient murine T cells (OVA53) exhibit defective proliferation control.^12^^,^^22^ Using the Fucci cell cycle reporter system (SA), the fraction of cells in G1 phase was estimated by measuring the number of mCherry-positive cells. While OVA53 cells vigorously proliferate in the absence of T cell receptor (TCR) signaling (Figure 2A, TCR (−)), about 70% of the cells were induced to be arrested at the G1 phase by T cell receptor (TCR)-mediated signaling in *Ambra1*-sufficient OVA53 cells (Figure 2A, TCR (+), Parent). However, only 7.6% of *Ambra1*-deficient OVA53 cells were in G1 phase after TCR-signaling (Figure 2A, *Ambra1* KO + Empty), suggesting that proliferation control was defective in the absence of AMBRA1. In order to examine whether the substitutions of AMBRA1 that appeared in patients with Cowden syndrome described above affected its function, AMBRA1 wild-type (WT), Cowden syndrome patient-type mutant (CS mut), and control empty vector were transfected into *Ambra1* deficient OVA53 cells. Enforced expression of human wild-type AMBRA1 in AMBRA1-deficient cells restored TCR-mediated cell-cycle arrest (Figure 2A, *Ambra1* KO + WT). However, the patient-type AMBRA1 mutant did not restore G1 arrest induction (Figure 2A, *Ambra1* KO + CS mut). Because Fucci is a system for visualizing the ubiquitin-mediated degradation of cell cycle-related proteins, it does not directly observe DNA replication associated with cell cycle progression. In order to further confirm the effect of AMBRA1 on cell cycle control, we performed another cell cycle analysis in which DNA replication in the S phase was estimated by BrdU incorporation. Conventional cell cycle analysis showed similar results (Figure S4A). Before transducing the TCR signal, more than 70% cells of the parent cells, as well as *Ambra1*-deficient cells transfected with either empty vector or CS mutant, incorporated BrdU to synthesize nascent DNA strands during the 30 min pulse (Figure S4A, Parent, *Ambra1* KO + Empty, *Ambra1* KO + CS mut). However, BrdU-incorporated cells were reduced to 64% in AMBRA1 WT over-expressing cells (Figure S4A, *Ambra1* KO + WT). This suggests that AMBRA1 suppresses entry into the S phase. While BrdU-incorporated cells (i.e., cells in S phase) were evidently reduced by TCR-signaling to 9 to14% in AMBRA1 WT-expressing cells (Figure S4A, Parent and *Ambra1* KO + WT), 35 to 40% cells remained to be in S phase in *Ambra1* deficient or CS mutant-expressing cells. These results suggested that the ability of AMBRA1 to induce cell-cycle arrest was impaired in patient-type AMBRA1 cells.

In contrast to MEFs expressing all D-type cyclins (cyclin D1, D2, and D3), OVA53 cells preferentially express cyclin D3. The transcription level of *cyclin d3* (*Ccnd3*) was high, whereas the expression levels of *cyclin d1* (*Ccnd1*) and *d2* (*Ccnd2*) were quite low (Figure S4B), presumably reflecting the characteristics of T lymphomas, such as Jurkat cells.^23^ *Ambra1*-deficient OVA53 cells expressed higher levels of cyclin D3 protein than the parental cells (Figure 2B), and the expressions of cyclinD1 and D2 were negligible. Because *cyclin d3* transcripts were lower in *Ambra1*-deficient cells (Figure S4B), it was suggested that AMBRA1 mainly plays a role at the post-transcriptional level. While the addition of WT AMBRA1 to *Ambra1*-deficient OVA53 cells decreased cyclin D3 expression, the patient-type mutant did not (Figure 2B, *Ambra1* KO + WT and + CS mutant). Similar results were obtained using *Ambra1*^*flox/flox*^ MEF culture system (Figure S3), although the impairment of CS mutant ability seemed to be modest when compared to that in the OVA53 cell culture system. In OVA53 cells, exogenous HA-tagged cyclin D3 was repressed by WT AMBRA1, but to a lesser extent by the CS mutant AMBRA1. CDK4 expression showed a similar trend; however, this difference was less prominent than that observed for cyclin D3 (Figure S4C). Mass spectrometry analysis of the co-immunoprecipitated proteins in OVA53 cells confirmed that Beclin1, cyclin D, CDK4, and CDK6 bind to AMBRA1, as previously reported (Figure S5 and Table S2). In addition, many ubiquitin-related proteins, including Cullin E3 ubiquitin ligases (Cullin-1, -2, -3, -4A, -4B, −5, and −7) and the adaptor protein damage-specific DNA-binding protein 1 (DDB1) were detected, suggesting that AMBRA1 is involved in controlling protein stability through the ubiquitin/proteasomal system (Figure S5).

In the presence of the proteasome inhibitor MG132, cyclin D3 expression did not differ between *Ambra1*-sufficient and -deficient cells, suggesting that AMBRA1 is involved in the proteasomal degradation of cyclin D, and that its function was attenuated in the CS mutant (Figure 2C). Furthermore, when *de novo* protein synthesis was inhibited by CHX, cyclin D3 levels rapidly decreased in AMBRA1 WT-expressing cells but were more stable in the absence of AMBRA1 and in the presence of the CS mutant (Figures 2D and 2E).

**Figure 3:**
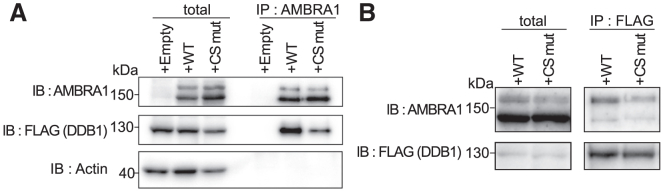
AMBRA1 mutant had a reduced ability to bind to DDB1 (A) FLAG-tagged DDB1 was introduced in *Ambra1*-disrupted OVA53 cells which had been transfected with either empty vector (+Empty), *AMBRA1* wild-type (+WT), or patient-type *AMBRA1* mutant (+CS mut). Cell lysates were subjected to immunoprecipitation (IP) using an anti-AMBRA1 antibody. The IP products were analyzed via Immunoblot to detect FLAG (DDB1). (B) Cell lysates were subjected to IP using an anti-FLAG antibody. The IP products were analyzed via Immunoblot to detect AMBRA1 WT and CS mutant.

Several groups in the past have reported that AMBRA1 serves as a ubiquitin E3 ligase for D-type cyclins via its interaction with DDB1-Cullin4A/4B (CRL4).^13^^,^^14^^,^^15^ Immunoprecipitation (IP) analysis was performed to examine binding affinity for DDB1 (Figure 3). WT AMBRA1 pulled down more FLAG-tagged DDB1 than did the CS mutant AMBRA1 (Figure 3A). Reciprocal FLAG-DDB1 immunoprecipitation confirmed that the binding of DDB1 to WT AMBRA1 was stronger than that to the CS mutant (Figure 3B). This suggested that the binding to DDB1 was impaired in the CS mutant AMBRA1. In contrast, binding to cyclin D3 was slightly enhanced (Figure S6). Therefore, it is suggested that the impaired binding of the CS mutant to DDB1-CRL4 leads to the de-repression of cyclin D expression, resulting in symptoms that appear in the family members of patients with Cowden syndrome.

### Increased animal size and enlarged organs of AMBRA1 conditional knockout mice

**Figure 4:**
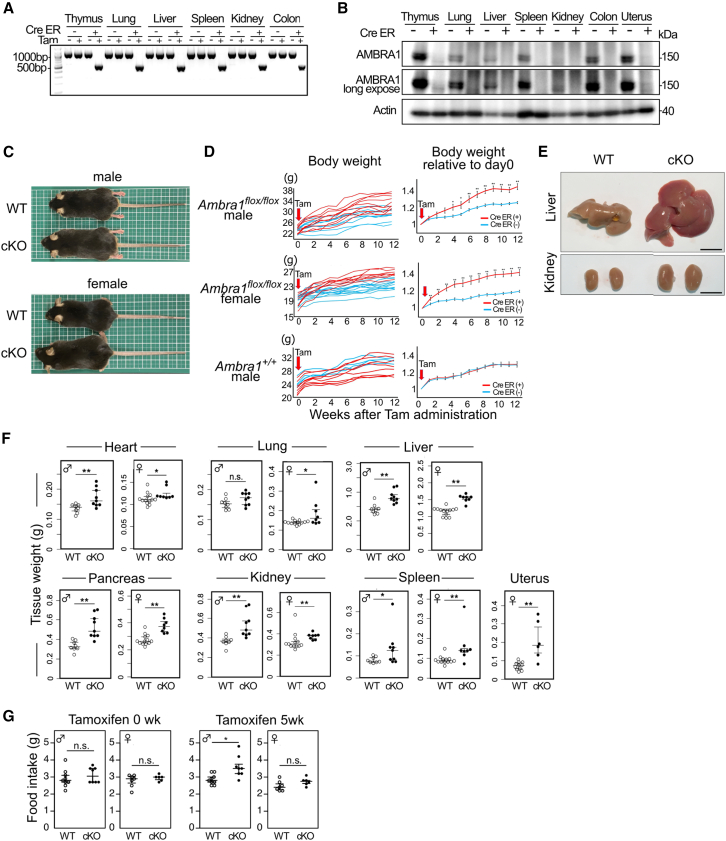
Marked increase in the body size and sizes of organs in *Ambra1* cKO mice (A) *Ambra1*^*flox/flox*^ mice were crossed with *Rosa-Cre-ERT2*-Tg mice. Tamoxifen (Tam) was administered to the mice at age 8 weeks to delete *Ambra1* exon 4 when the mice possessed Cre-ERT2 transgene (Cre-ER+). PCR primers for amplifying deleted/undeleted segments are indicated as arrows shown in Figure S2A (5′F and 3′R). DNA from various organs was subjected to PCR analysis, and the amplified undeleted segment and deleted allele of 1097 bp and 576 bp, respectively, were detected. (B) Ubiquitously expressed AMBRA1 was diminished in *Ambra1* cKO mice. Lysates of the thymus, lung, liver, spleen, kidney, colon, and uterus of *Ambra1*^*flox/flox*^*Cre-ERT2* (+) and *Ambra1*^*flox/flox*^*Cre-ERT2* (−) mice (Cre-ER+ and Cre-ER-, respectively), which were administered with Tam were subjected to immunoblot analysis. (C) Body size of TAM administered-*Ambra1* cKO mice and TAM administered-littermates (WT) at 31 weeks of age. (D) Body weights of *Ambra1* cKO (Cre-ER(+), red lines) and control mice (Cre-ER(−), blue lines) after Tam administration at 7–9 weeks of age (Top: male, middle: female). Body weights of male *Ambra1*^*+/+*^ mice with and without Rosa- Cre-ERT2 are shown as controls (bottom). Individual value (left) and mean value ±standard error (SE) of body weights relative to that at the start point (week 0) are indicated (right). (*Ambra1*^*flox/flox*^ male Cre-ER (+): *n* = 10, *Ambra1*^*flox/flox*^ male Cre-ER(−): *n* = 8, *Ambra1*^*flox/flox*^ female Cre-ER(+): *n* = 8, *Ambra1*^*flox/flox*^ female Cre-ER(−): *n* = 13, *Ambra1*^*+/+*^ male Cre-ER(+): *n* = 8, and *Ambra1*^*+/+*^ male Cre-ER(−): *n* = 4) (∗*p* < 0.05 and ∗∗*p* < 0.01 in the Student’s t test). (E) The liver and kidneys were larger in TAM administered-*Ambra1* cKO mice compared to those in TAM administered-littermate control (female littermates, 12 months old). Scale bars, 1 cm. (F) The weights of the heart, lungs, liver, pancreas, kidney, spleen, and uterus of the mice shown in (D). (TAM administered-*Ambra1* cKO (cKO, *n* = 8 and 9, female and male, respectively) and TAM administered-control mice (WT, *n* = 13 and 8, female and male, respectively)). Data are presented as medians with 25^th^–75^th^ percentiles. (∗*p* < 0.05, ∗∗*p* < 0.01, and n.s.: not significant in the Mann-Whitney U-test). (G) The daily average food intake of *Ambra1* cKO (cKO, *n* = 6 and 8, female and male, respectively) and control mice (WT, *n* = 7 and 8, female and male, respectively). Food intake was measured before Tamoxifen injection (Tamoxifen 0 weeks) and after 5weeks Tamoxifen injection (Tamoxifen 5 weeks). Data are presented as medians with 25^th^–75^th^ percentiles. (∗*p* < 0.05 and n.s.: not significant in the Mann-Whitney U-test).

As described above, we generated drug-inducible *Ambra1* conditional knock out mice (*Ambra1* cKO mice). After the mice were 8 weeks old, Tam was administered to induce the deletion of *Ambra1* exon 4 in *Ambra1*^*flox/flox*^ *Rosa- Cre-ERT2-Tg* mice (Cre ER+ and Tam+, Figure 4A), thereby abolishing AMBRA1 protein expression (Cre ER +, Figure 4B), which was ubiquitously expressed in Tam-administered control mice (Cre ER-). However, an exception was observed in the brain, where exon 4 deletion was incomplete (data not shown), presumably because of inefficient Tam delivery. Surprisingly, the body size of *Ambra1* cKO mice was larger than that of control mice (Figure 4C). *Ambra1* cKO mice grew faster than the control mice (Figure 4D). Ten weeks after *Ambra1* deletion, the body weights of male and female mice increased by approximately 45% and 30%, respectively, whereas the values in the control group were 20% and 10%, respectively (Figure 4D). The sizes of the liver and kidneys (Figure 4E) and the weights of different organs (Figure 4F), including the heart, lungs, liver, pancreas, kidneys, spleen, and uterus, were significantly increased in *Ambra1* cKO mice compared with those of control mice. There was no difference in food intake between the control mice and *Ambra1* cKO mice before Tam administration. Five weeks after Tam administration, food intake in male *Ambra1* cKO mice increased significantly. There was also a tendency toward food intake in female *Ambra1* cKO mice, which was not significant (Figure 4G).

**Figure 5:**
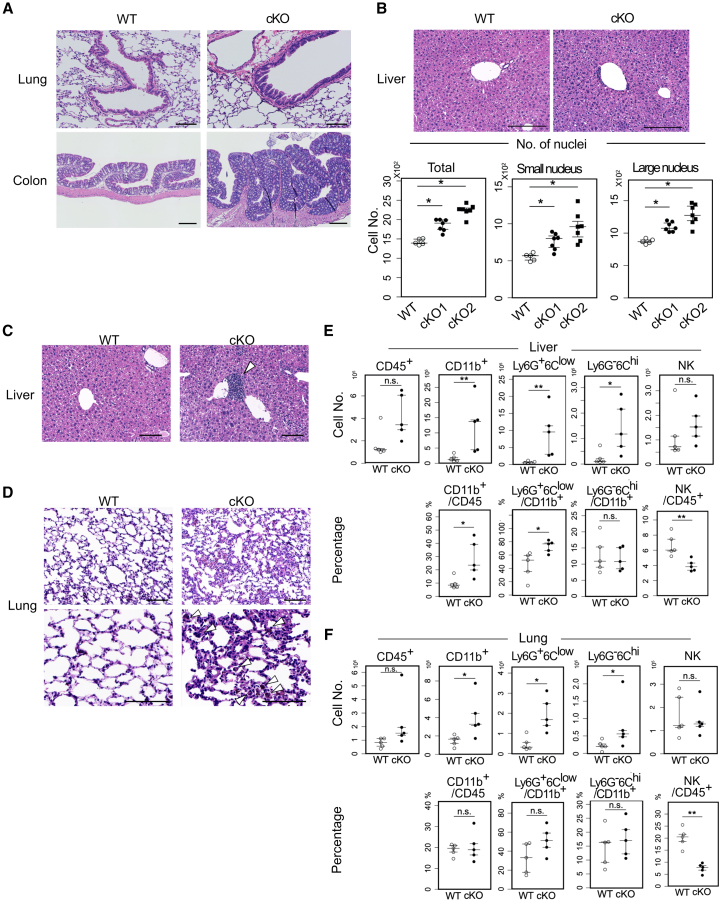
Histological manifestations of *Ambra1* cKO mice (A) Hematoxylin and eosin (H&E) stained sections of the lung (top) and colon (bottom). Papillary growth of the bronchus (upper right) and hyperplasia of the colon epithelium (lower right) in *Ambra1* cKO mice are presented. Scale bars, 100 μm (lung) and 200 μm (colon). (B) H&E-stained sections of the liver (upper). Scale bars, 200 μm. The numbers of cell nuclei per microscopic field (lower, WT: *n* = 5, cKO1: *n* = 8, and cKO2: *n* = 8) were calculated by ImageJ software. The signals from the nuclei were divided into two groups: <15.5 μm^2^ (small nucleus) and >15.5 μm^2^ (large nucleus). Small nuclei mainly corresponded to non-parenchymal cells, such as blood-derived cells, endothelial cells, and stellate cells, while large nuclei corresponded to hepatocytes. Data are presented as medians with 25^th^–75^th^ percentiles. (∗*p* < 0.05 in the Steel–Dwass test). (C and D) Hematoxylin and eosin (H&E)-stained sections of the liver (C) and lung (D) of WT and *Ambra1* cKO mice. Infiltration of hematopoietic cells was frequently observed (C, D, arrowheads). Scale bars, 200 μm (C), 100 μm [(D) upper], and 50 μm [(D) bottom]. (E and F) FACS analysis of hematopoietic cells in the liver (E) and lung (F). Eight months after Tam administration, female mice were used for the experiment. After collagenase treatment and Percoll gradient centrifugation, fractions enriched in hematopoietic cells were analyzed by FACSverse. The number of CD45^+^, CD11b^+^, Ly6G^+^Ly6C^low^, Ly6G^−^Ly6C^hi^, and NK1.1^+^ cells in the liver (E, upper) and lungs (F, upper) is shown. The proportions of CD11b^+^ cells in CD45^+^ cells, Ly6G^+^Ly6C^low^ cells in CD11b^+^ cells, Ly6G^−^Ly6C^hi^ cells in CD11b^+^ cells, and NK1.1^+^ cells in CD45^+^ cells are shown (E and F, bottom). Data are presented as medians with 25^th^–75^th^ percentiles. (∗*p* < 0.05, ∗∗*p* < 0.01, and n.s.: not significant in the Mann-Whitney U-test).

Histological manifestations were observed in *Ambra1* cKO mice. Papillary growth of the bronchial epithelium and hyperplasia of the colon epithelium in *Ambra1* cKO mice are shown (Figure 5A). Although these manifestations were not evident in the liver, the average number of cell nuclei per microscopic field was significantly higher in *Ambra1* cKO mice than in control mice (Figure 5B). Nuclear signals were divided into two groups using the ImageJ software (NIH), where small nuclei mainly corresponded to non-parenchymal cells, such as blood-derived, endothelial, and stellate cells, whereas large nuclei corresponded to hepatocytes. The signals for both groups were increased in the livers of *Ambra1* cKO mice (Figure 5B, bottom). Therefore, the increased organ size in *Ambra1* cKO mice probably resulted from an increase in cell number and not from an increase in the cell size of both parenchymal and non-parenchymal cells. Consistent with this, the cell size of *Ambra1*-deficient MEFs was found to be smaller than that of parental MEFs (Figure S7), whereas *Ambra1*-deficient MEFs proliferated more rapidly than *Ambra1*-sufficient MEFs (Figure 1C), further supporting the notion that Ambra1 loss promotes cell proliferation rather than hypertrophy.

Furthermore, hematopoietic cells frequently infiltrated the perivascular areas of the liver and pulmonary alveoli in *Ambra1* cKO mice (Figures 5C and 5D). Then, blood-derived cells from the liver and lungs were collected, enriched by collagenase treatment and Percoll gradient centrifugation, and analyzed using flow cytometry (Figures 5E and 5F). Among the CD45^+^ hematopoietic cells, CD11b^+^Gr- 1(Ly6C/6G)^+^ myeloid lineage cells increased in *Ambra1* cKO mice. To examine whether the infiltrating hematopoietic cells were intrinsically affected by *Ambra1* deficiency, bone marrow (BM) cells obtained from *Ambra1*^*flox/flox*^*-Rosa-Cre-ERT2- Tg*(+) and *control -Rosa-Cre-ERT2-Tg*(−) mice (*Ambra1* cKO-BM cells and control BM cells, respectively) were transferred to irradiated syngeneic normal mice, followed by Tam administration. We found that the red blood cell (RBC) count was predominantly low for several weeks after the transplantation of *Ambra1* cKO-BM cells, whereas the white blood cell (WBC) count did not differ from that of the control during this period (Figure S8A). However, after 10–13 weeks, the WBC count was lower than that in the control group. Flow cytometric analysis of peripheral blood mononuclear cells (PBMC) revealed that B220^+^ cells were prominently reduced, whereas Gr1^+^(both CD11b^+^ and CD11b^−^) cells were increased (Figures S8B and S8C). These results suggest that *Ambra1*-deficient hematopoietic cells are intrinsically affected and skewed to differentiate into myeloid lineage cells such as Gr-1^+^ cells, but are less skewed to produce B cells. Additionally, an extremely large number of peripheral white blood cells, consisting of mature neutrophils with polysegmented nuclei and immature neutrophils with toroidal nuclei, were observed in some *Ambra1* cKO BM-transferred mice (Figure S8D). Prominent blood cell infiltration (Figure S8E, right) and white masses, consisting mainly of CD11b^+^Gr-1^+^ cells, were observed in the liver (Figure S8E, left arrowheads). The masses formed follicular-like structures, suggesting that the infiltrating cells had proliferated. However, the exome analysis of these masses did not reveal any common mutations (data not shown). Collectively, *Ambra1*-deficient BM cells tended to differentiate into myeloid cell lineages that could infiltrate organs such as the lungs and liver and proliferate.

### Hyper expression of D-type cyclins with enhanced cell proliferation in AMBRA1 conditional knockout mice

**Figure 6:**
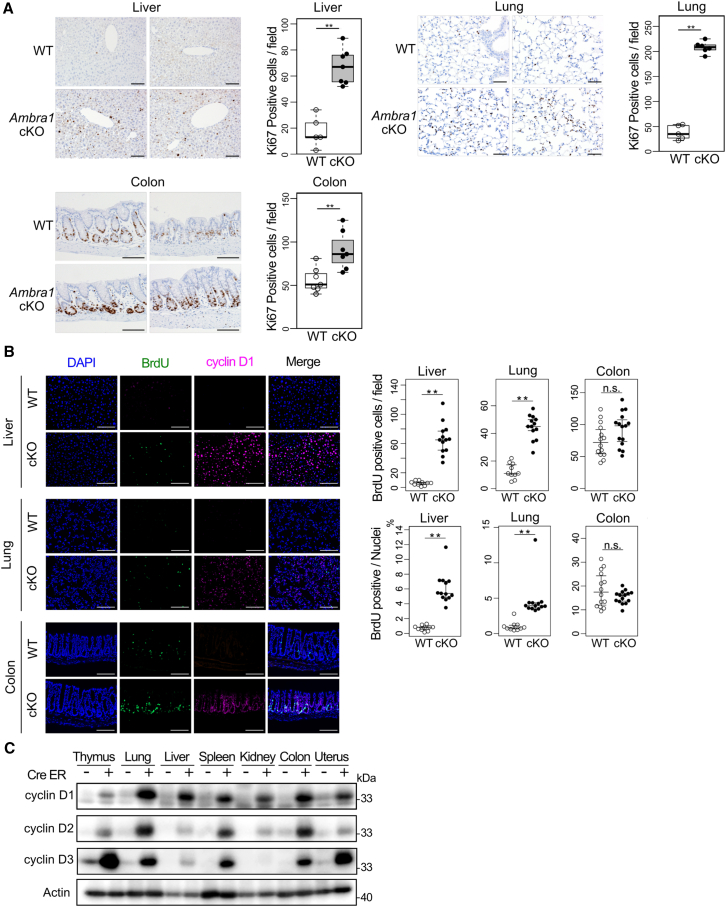
Enhanced cell proliferation with increased cyclin D expression in *Ambra1* cKO mice (A) Two weeks after Tam administration, proliferating cells in the liver (top), lung (right), and colon (bottom) were detected by Ki-67 expression. Ki-67 positive cells were counted, and signal counts per field were calculated using the ImageJ program. Scale bar, 100μm. Data are presented as medians with 25^th^–75^th^ percentiles. (∗∗*p* < 0.01 in the Mann-Whitney U-test). (B) The lung, liver, and colon of *Ambra1* cKO (cKO) and littermate control (WT) mice, which had been intraperitoneally injected with BrdU 24 h before analysis and subjected to immunohistochemical analysis of BrdU (green) and cyclin D1 (magenta) using fluorescence microscopy. BrdU^+^ cells per microscopic field (upper right) and BrdU-stained nuclei per total nuclei (lower right) were calculated using the ImageJ program. Scale bar, 100 μm. Data are presented as medians with 25^th^–75^th^ percentiles. (∗∗*p* < 0.01, and n.s.: not significant in the Mann-Whitney U-test). (C) Expression of cyclin D proteins extracted from the thymus, lung, liver, spleen, kidney, colon, and uterus of *Ambra1*^*flox/flox*^-*Rosa-Cre-ERT2-Tg (−)* (Cre-ER-) and *Ambra1*^*flox/flox*^-*Rosa-Cre-ERT2-Tg (+)* (Cre-ER+) mice subcutaneously injected with TAM. AMBRA1 shown in Figure 4B and cyclin D1 shown in (C) were detected simultaneously on the same membrane, while cyclin D2 and cyclin D3 was detected after reprobing. The same Actin blot is shown in both figures as the loading control.

Ki-67 staining revealed that cell proliferation was evidently increased in the liver, lungs, and colon of *Ambra1* cKO mice that had been administered Tam 2 weeks prior (Figure 6A). Similarly, BrdU-incorporated cells during 24 h increased in the liver and lungs of *Ambra1* cKO mice, indicating an increase in the number of cells entering the S phase (Figures 6B and S9, green). Interestingly, in the colon, the localization of BrdU^+^ cells was distinct between *Ambra1* cKO mice and WT mice. In WT mice, BrdU^+^ cells were mainly observed above the crypt bottom, where “transit amplifying (TA)” cells move upward and proliferate. In *Ambra1* cKO mice, BrdU^+^ cells were mostly observed at the bottom of the crypt, where crypt-based columnar cells (CBC) and Paneth-like cells were located. These observations suggest that AMBRA1 suppresses S phase entry and contributes to cell cycle exit in certain cell lineages and differentiation stages.

As expected, the number of cyclin D-expressing cells dramatically increased in *Ambra1* cKO mice (Figures 6B and S9, magenta). It has been suggested that enhanced cyclin D expression promotes entry into the S phase. Enhanced expression of D-type cyclins was observed in various organs, including the thymus, lung, liver, spleen, kidney, colon, and uterus (Figure 6C). While the protein levels of cyclins D1, D2, and D3 were greatly enhanced, *cyclin d* (*Ccnd*) transcript levels did not increase. In some instances, a decrease in these levels was observed (Figure S10 and Table S3). Therefore, the increased expression of cyclin D mainly results from post-transcriptional regulation.

### AMBRA1 conditional knockout mice were susceptible to malignant tumors

**Figure 7:**
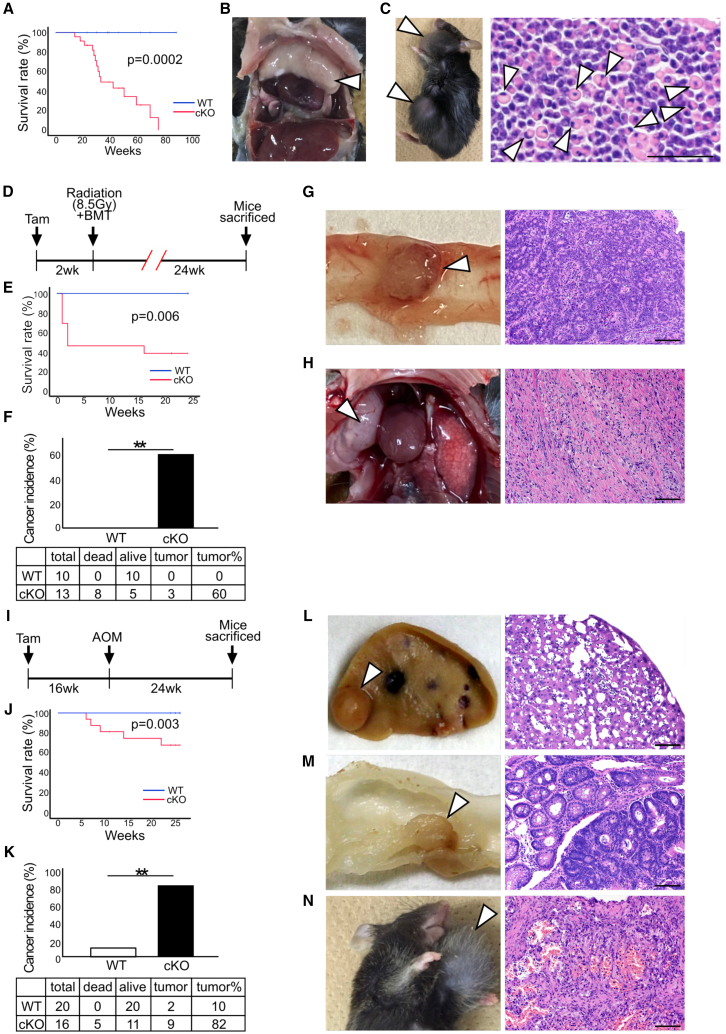
Increased susceptibility of *Ambra1* cKO mice to tumorigenesis (A) Kaplan-Meier survival curve of *Ambra1* cKO and control littermates (WT) after Tam administration. *p* value was assessed using the log rank test. (B and C) Spontaneous tumors in *Ambra1* cKO mice. (B) Thymus hypertrophy (arrowhead) because of CD4^+^CD8^+^ T lymphoma, leading to lethal respiratory distress. (C) Hypertrophies of the submandibular and inguinal lymph nodes (left, arrowheads). Hematoxylin and eosin (H&E)-stained section of the submandibular lymph node showed signet ring-like cells (right, arrowheads), reflecting the secretory function of immunoglobulin. Scale bar, 50 μm. (D–H) Irradiation-induced tumors in *Ambra1* cKO mice transferred with *Ambra1*-sufficient hematopoietic cells. (D) Experimental schedule: Two weeks after tamoxifen administration, *Ambra1*^*flox/flox*^ mice with or without Rosa-Cre-ERT2-Tg were irradiated with 8.5 Gy X-ray, and 1×10^6^ WT bone marrow cells were transferred. The mice were examined for carcinogenesis after 24 weeks. (E) Kaplan-Meier Survival curves of *Ambra1* cKO and control littermates (WT) after BMT. *p* value was assessed using the log rank test. (F) The percentage of cancer incidence in irradiated *Ambra1* cKO and control mice. (∗∗*p* < 0.01 by Chi-square method). (G) A flat polyp without a stalk in the colon of an *Ambra1* cKO mouse with WT blood cells. The proliferation of atypical columnar epithelium having a large nucleus with tubular and cribriform structure indicated adenocarcinoma. (H) A round tumor (7 mm) with a smooth surface in the thoracic space of an *Ambra1* cKO mouse with WT blood cells. Histologically, a diffused proliferation of tumor cells having an oval or spindle nucleus with abundant collagen fibers was observed. The lymphocytes were intermingled. (I–N) AOM-induced cancer in *Ambra1* cKO mice. (I) Experimental schedule: *Ambra1* cKO and control mice were intraperitoneally injected with AOM, and carcinogenesis was examined 25 weeks later in the surviving mice (cKO: 11 and WT: 20). (J) Kaplan-Meier survival curves of *Ambra1* cKO and control littermates (WT) after AOM injection. *p* value was assessed using the log rank test. (K) The percentage of cancer incidence in *Ambra1* cKO and control mice injected with AOM. (∗∗*p* < 0.01 by Chi-square method). AOM-induced tumor in the liver (L) and colon (M) of *Ambra1* cKO mice. (N) AOM-induced angiosarcoma in *Ambra1* cKO mice.

The lifespan of the *Ambra1* cKO mice was relatively short. A few mice survived for >80 weeks after Tam administration (Figure 7A). This increased mortality rate may have resulted from at least in part the frequent occurrence of malignant lymphomas. In the case of thymic lymphoma, which was frequently observed in *Ambra1* cKO mice, the enlarged thymus compressed the lungs and caused lethal respiratory distress (Figure 7B, arrowhead). Hypertrophic lymph nodes, mainly composed of CD19^+^ B-cell lymphoma cells, are another example of tumor susceptibility (Figure 7C, arrowhead).

As mentioned above, the number of myeloid cells increased, and they infiltrated the organs of *Ambra1* cKO mice (Figure 5C). Inflammation and immune dysregulation have been reported to contribute to tumor initiation, progression, and metastasis.^24^^,^^25^^,^^26^ For example, some myeloid cells such as those expressing CD11b and Gr-1 can suppress immune responses in tumor tissues.^27^ Therefore, susceptibility to tumorigenesis in *Ambra1* cKO mice may result from enhanced inflammation and/or immune suppression. To exclude the contribution of *Ambra1* deficiency to hematopoietic cells, WT BM cells were transferred to *Ambra1* cKO and control mice after X-ray irradiation (Figures 7D–7H). Polymerase chain reaction (PCR) analysis indicated that almost all spleen cells were replaced by the transferred *Ambra1* - sufficient cells (Figure S11A). During the 24 weeks after BM transfer, 72% of *Ambra1* cKO mice with WT hematopoietic cells died, whereas all control mice survived (Figure 7E), suggesting that *Ambra1* cKO mice were more sensitive to irradiation. Among the surviving *Ambra1* cKO mice, 60% (three out of five) developed malignant tumors, whereas no tumors were found in the control mice (Figures 7F–7H, S11B, and S11C). These observations indicate a high extent of carcinogenesis in *Ambra1* cKO mice aside from the dysregulation of blood-derived cells. Therefore, AMBRA1 may suppress tumorigenesis in a cell-autonomous manner. However, some effects on the tumor-extrinsic environment formed by blood-derived cells cannot be excluded.

To further examine susceptibility to tumorigenesis, a carcinogenic substance, azoxymethane (AOM), was administered to *Ambra1* cKO mice (Figures 7I–7N). AOM induces colon cancer in combination with dextran sulfate sodium (DSS) in drinking water, causing an inflammatory response in the colon.^28^ However, we administered only AOM. This relatively mild treatment led to tumor development in only 10% (2 out of 20) of the WT mice (Figure 7K). However, 82% (9 out of 11) of the surviving *Ambra1* cKO mice developed prominent tumors in various organs, such as the liver (Figures 7L and S11D) and colon (Figures 7M and S11E), and lymph and blood vessels (Figure 7N).

Genomic analyses revealed that spontaneous or AOM-induced tumors in *Ambra1* cKO mice were composed of cells harboring common mutations that were not observed in the control DNA. Among these, 4–29 missense mutations were detected in tumors (Table S4). The mutant alleles accounted for an average of 31.9%, suggesting that only a limited number of cells underwent clonal expansion to form tumors. Notably, mutations in the cancer-associated genes KRAS G12D (spontaneous thymic T lymphoma) and Ctnnb1 G34E (AOM-induced colon cancer) were detected in two of the four tumors. These findings are consistent with the symptoms of patients with Cowden/Cowden-like syndrome who experience a high risk of malignancy. It is plausible that enhanced cell cycle activity caused by AMBRA1 dysfunction promotes genomic instability, resulting in malignancy. AMBRA1 may suppress the proliferation of cancer driver mutation-harboring cells. Collectively, these results strongly suggested that AMBRA1 serves as a tumor suppressor *in vivo* condition.

## Discussion

AMBRA1 is a substrate receptor of the ubiquitin-conjugating system and serves as a tumor suppressor. Several substrates, such as BECLIN1, ULK1 (Unc-51-like autophagy activating kinase-1) and D-type cyclins, have been reported to be ubiquitinated by AMBRA1.^7^^,^^8^^,^^13^^,^^14^^,^^15^ While the ubiquitination of BECLIN1 and ULK1 by AMBRA1 has been reported to be mediated by a specific RING finger E3 ubiquitin ligase, TRAF6 (tumor necrosis receptor-associated factor 6),^8^ D-type cyclin ubiquitination is mediated by the Cullin4A/B-RING E3 ligase complex (CRL4).^13^^,^^14^^,^^15^ Previously, we reported that missense mutations in *AMBRA1* are strong candidates for familial tumor Cowden syndrome.^6^ In this study, we confirmed that the mutant attenuated binding to DDB1, resulting in impaired control of cyclin D expression and cell cycle exit.

DDB1, a CRL4 component, is an adaptor protein between CRL4 and AMBRA1.^9^ Recently, the techniques of hydrogen deuterium exchange mass spectrometry (HDX-MS) and cryo-electron microscopy (cryo-EM), the structure of AMBRA1 in complex with DDB1 was proposed.^29^ According to the study, AMBRA1 has a split WD40 domain formed by its N- and C- terminal regions (residues 41–204 and 853–1044, respectively) which comprises a β-propeller architecture with 7 repeats. The authors reported that in addition to the WD40 repeat, the N-terminal helix-loop-helix (residues 1–39) is critical for AMBRA1 binding to DDB1. The CS mutant described in our study harbors a Q30R substitution in the N-terminal helix-loop-helix region, which results in a change from a negative to a positive charge for Q30. We found that binding to DDB1 was decreased in the CS mutant. It is reasonable that the substitution affected binding to DDB1, resulting in the attenuated function of cyclin D control. The other substitution in the CS mutant was R1195S. Residue 1195 is located outside the C-terminal WD40 domain and consists of intrinsically disordered regions (IDRs) that lack recognizable protein motifs. The significance of these substitutions should be explored further in future studies.

We generated *Ambra1* cKO mice that exhibited hyperproliferation in various organs and were susceptible to spontaneous or chemicalirradiation-induced tumorigenesis. As expected, cyclin D expression was markedly higher in various organs of *Ambra1* cKO mice. The high activity of CDK4/6 in the early G1 phase or even in the G2 phase of the mother cell can determine cell cycle progression,^16^^,^^17^^,^^18^ and enhanced CDK4/6 activity by derepressing cyclin D expression may result in the failure of cell cycle exit and an increase in the number of cells entering the S phase.

In the colon, the number of BrdU^+^ cells was increased in cKO mice at the crypt bottom, where the CBC was interspersed between Paneth-like cells and intestinal stem cells.^30^ However, in TA cells that reside above the crypt bottom, BrdU incorporation did not increase despite the prominent expression of cyclin D. Cell division at the crypt bottom may be controlled by the AMBRA1-cyclin D axis, whereas TA cells may proliferate in a cyclin D-independent manner. It has been suggested that AMBRA1 depletion enhances cell proliferation in a lineage/differentiation stage-specific manner. Rapid cycling or quiescent intestinal stem cells reside near the bottom of the crypts.^31^^,^^32^ Further studies are required to elucidate the effects of AMBRA1 deficiency on the proliferation and quiescence of intestinal stem and progenitor cells.

Among the hematopoietic cells, the number of CD11b^+^Gr1^+^ myeloid cells increased and infiltrated both the lungs and liver. Bone marrow (BMT) transfer experiments have shown that BM cells are skewed toward differentiating into myeloid cells. However, the number of erythroid- and B-lineage cells decreased. Surprisingly, in some BMT experiments, CD11b^+^Gr1^+^ cells proliferated vigorously to form massive clumps in the liver, without undergoing malignant transformation. Interestingly, similar results were previously reported in autophagy factor *Atg7* deficient mice.^33^ In this study, hematopoietic cell-specific deletion of Atg7 led to the accumulation of myeloid lineage cells, and massive clumps composed of myeloid cells were frequently observed in the liver. Since AMBRA1 is known to be involved in autophagy progression,^34^ it is possible that defective autophagy resulting from AMBRA1 deletion leads to perturbed hematopoietic cell differentiation and proliferation.

Similar to the patients with Cowden/Cowden-like syndrome, *Ambra1* cKO mice were susceptible to malignant tumors, some of which contained hotspot mutations in Kras (G12D) and Ctnnb1 (G34E) (Table S4). The enhanced cell proliferation resulting from *Ambra1* depletion may increase the risk of somatic cell mutations. In particular, derepressed cyclin D expression in S phase has been reported to inhibit DNA mismatch repair, thus resulting in genomic instability.^15^^,^^35^ Alternatively, AMBRA1 may contribute to the growth suppression of cells harboring cancer-associated mutations, which are found even in normal tissues, to maintain homeostasis.^36^^,^^37^^,^^38^^,^^39^^,^^40^ We observed that AMBRA1 suppressed the growth of embryonic fibroblasts expressing active H-RAS G12V. Although we have focused on cyclin D expression in association with S entry in this study, it is plausible that AMBRA1 has other target molecules, as well as autophagy-related molecules, that may play a role in controlling cell proliferation and tumor growth. It is well known that autophagy suppresses tumorigenesis and also promotes tumor growth through removing dangerous damaged organelles and through acquiring chemoresistance, respectively.

In addition to CRL4, other E3-ligases such as CRL1, 3, 2, and 7, were detected in the AMBRA1 co-immunoprecipitated molecules. Various transcription- and translation-related molecules were detected (Table S2). Recently, it was reported to AMBRA1 regulates the translation of FAS-related molecules, thereby modulating T cell apoptosis following TCR stimulation.^41^ How AMBRA1 contributes to the integrity of the living body requires further investigation. Given AMBRA1’s broad role in maintaining vital cellular processes, it is likely that this protein also has unknown functions. Further investigations into AMBRA1 are essential to uncover its potential contributions to unresolved biological phenomena, paving the way for deeper insights into life sciences.

### Limitations of the study

This study did not investigate the autophagic function of the AMBRA1 CS mutant (Q30R, R1195S), leaving its potential impact on autophagy pathways unresolved. Given that AMBRA1 is a key regulator of autophagy, these mutations may alter autophagic activity. Furthermore, *in vivo* validation using knock-in mice harboring the Q30R and R1195S mutations was not conducted. Such models are essential for elucidating the physiological relevance of these mutations in tissue homeostasis and tumorigenesis. Future studies should investigate the autophagic role of the AMBRA1 CS mutant and assess its *in vivo* effects using knock-in mouse models.

## Resource availability

### Lead contact

Further information and requests for resources and reagents should be directed to and will be fulfilled by the lead contact, Takehito Sato (stakehito@soka.ac.jp).

### Materials availability

All stable materials and reagents generated in this study are available from the lead contact upon request under a universal material transfer agreement.

### Data and code availability

• Sequencing data generated in this study have been deposited in DDBJ, and the accession number is listed in the key resources table.
• This article does not report original code.
• Any additional information required to reanalyze the data reported in this work article is available from the lead contact upon request.

## Acknowledgments

We thank Dr. Motoya Katsuki and Dr. Toshikuni Sasaoka for generously providing cA-H-RAS transgenic mice. This work was supported by 10.13039/501100001691JSPS KAKENHI (grant numbers JP19K16755 and 16H06279 [PAGS]), 10.13039/501100010656Tokai University School of Medicine Project Research (2017–18), and 10.13039/501100010656Tokai University School of Medicine Research Aid (2019–20). The authors thank the Medical Science College Office, Tokai University, for technical assistance. We would like to thank Editage (www.editage.jp) for English language editing.

## Author contributions

Conceptualization, H.A. and T.S.; methodology, H.A. and H.H.; formal analysis, H.A., T.K., K.M., Y.I., C.O-Y., Y.O., M.T., T.T., H.N., K.H., Y.H., M.H., Y.S., Y.N., K.H., A.A.I., T.Y., N.N., and H.H.; investigation, H.A., T.K., K.M., M.T., C.L., T.T., H.N., K.H., Y.N., K.H., A.A.I., T.Y., and H.H.; resources, T.S., K.H., M.O., H.H., and I.I.; data curation, M.T. H.N., K.H., Y.H., and M.H.; writing original draft, H.A. and T.S.; visualization, H.A., T.K., K.H., and H.H.; supervision, T.S.; project administration, T.S.; funding acquisition, H.A., T.S., I.I., and T.S.; writing review and editing, all authors.

## Declaration of interests

The authors declare no conflicts of interest.

## STAR★Methods

### Key resources table

**Table:** 

REAGENT or RESOURCE	SOURCE	IDENTIFIER
**Antibodies**		

anti-hamster IgG	Abcam	ab5738; RRID: AB_956019
anti-mouse CD3ε antibody (clone: 145-2C11)	BioLegend	100340; RRID: AB_2616674
APC anti BrdU antibody (clone: 3D4)	BioLegend	Cat# 364107; RRID: AB_2566451
Anti-BrdU	BD Biosciences	Cat# 555627; RRID: AB_395993
anti-Ambra1	CST	Cat# 24907S; RRID: AB_2798888
anti-Ki-67 (clone: D3B5)	CST	Cat# 9129T; RRID: AB_2687446
anti-cyclin D1 (clone:92G2)	CST	Cat# 2978S; RRID: AB_2259616
anti-cyclin D2 (clone:D52F9)	CST	Cat# 3741S; RRID: AB_2070685
anti-cyclin D2	Proteintech	Cat# 10934-1-AP; RRID: AB_2275319
anti-cyclin D3 (clone:18B6-10)	Santa Cruz	Cat# sc-453; RRID: AB_627353
anti-CDK4 (clone:DCS-35)	Santa Cruz	Cat# sc-23896; RRID: AB_627239
anti-CDK6 (clone:DCS-83)	CST	Cat# 3136T; RRID: AB_2229289
anti-Ras	CST	Cat# 3965S; RRID: AB_2180216
anti-HA (clone:12AC5)	Sigma-Aldrich	Cat# 11583816001; RRID: AB_514505
anti-HA	Proteintech	Cat# 51064-2-AP; RRID: AB_11042321
anti-α-tubulin (clone:DM1A)	Sigma-Aldrich	Cat# T6199; RRID: AB_477583
anti-β-actin (clone:AC-15)	Sigma-Aldrich	Cat# A5441; RRID: AB_2766243
Alexa 488 anti rabbi IgG	Thermo Fisher Scientific	Cat# A-11070; RRID: AB_2534114
Alexa 594 anti rabbi IgG	Thermo Fisher Scientific	Cat# A-11072; RRID: AB_2534116
HRP anti mouse IgG	Cytiva	Cat# NA9310-1mL; RRID: AB_772193
HRP anti rabbit IgG	Thermo Fisher Scientific	Cat# 10710965; RRID: AB_772191
HRP anti rat IgG	CST	Cat# 7077; RRID: AB_10694715
HRP anti rat IgG	Proteintech	Cat# SA00001-15; RRID: AB_28643409
FITC-*anti*-mouse CD45 (clone: 30F11)	BioLegend	Cat# 103107; RRID: AB_312972
PerCP/Cyanin5.5-*anti*-mouse CD11b (clone: M1/70)	BioLegend	Cat# 101227; RRID: AB_893233
PE/Cyanine7-*anti*-mouse Ly6C (clone: HK1.4)	BioLegend	Cat# 128017; RRID: AB_1732093
APC/Cyanine7-*anti*-mouse Ly6G (clone: 1A8)	BioLegend	Cat# 127623; RRID: AB_10640819
APC-*anti*-mouse NK1.1 (clone: PK136)	BioLegend	Cat# 108709; RRID: AB_313396
PE-*anti*-mouse TCRβ (clone: H57-597)	Thermo Fisher Scientific	Cat# 12-5961-82; RRID: AB_466066
PE/Cyanine7-*anti*-mouse CD4 (GK 1.5)	BioLegend	Cat# 100421; RRID: AB_312706
APC/Cyanine7-*anti*-mouse CD8α (clone: 53-6.7)	BioLegend	Cat# 100714; RRID: AB_312753
PE-*anti*-mouse CD45R/B220 (clone: RA3-6B2)	BD Biosciences	Cat# 553090; RRID: AB_394620
anti-mouse CD16/32 (clone: 93)	Thermo Fisher Scientific	Cat #14-0161-82; RRID: AB_467133
PE/Cyanine7-*anti*-human CD271 (NGFR) (clone: ME20.4)	BioLegend	Cat# 345110; RRID: AB_11203542
anti-Flag M2 agarose	Sigma-Aldrich	Cat# A2220; RRID: AB_10063035

**Bacterial and virus strains**		

ECOSTM Competent *E. coli* DH5 α	NIPPON GENE	Cat# 310-06236

**Biological samples**		

DNA of Blood mononuclear cells collected from the patient’s family members	This paper	N/A
DNA of Tumor tissue from wild-type mice and *Ambra1* cKO mice	This paper	N/A
RNA of liver and colon from wild-type mice and *Ambra1* cKO mice	This paper	N/A

**Chemicals, peptides, and recombinant proteins**		

Tamoxifen	Cayman Chemical Company	Cat# 13258
Corn oil	Sigma-Aldrich	Cat# C8267
5-Bromo-2′-deoxyuridine (BrdU)	BD Biosciences	Cat# 550891
Azoxymethane	Fujifilm	Cat# 011-20171
RPMI1640	Nissui	Cat# 5918
DMEM	Nissui	Cat# 5919
FBS	Sigma-Aldrich	Cat# 173012-500ML
HEPES	Sigma-Aldrich	Cat# H3375-250G
sodium pyruvate	Sigma-Aldrich	Cat# P2256-100G
Glutamate	Fujifilm	Cat# 072-00523
2-mercaptoethanol	GIBCO	Cat# 21985023
penicillin/streptomycin	Sigma-Aldrich	Cat# P4333
GENETICIN	GIbco	Cat# 11811-031
Puromycin	Fujifilm	Cat# 160-23151
Trypsin-EDTA	Sigma-Aldrich	Cat# T3924
Opti-MEM reduced serum Medium	Thermo Fisher Scientific	Cat# 31985070
4-OHT	Cayman Chemical Company	Cat# 68392-35-8
CELLBANKER 1	Takara bio	Cat# CB011
XtremeGENE 9 DNA Transfection Reagent	Roche	Cat#XTG9-RO
PFA	Fujifilm	Cat# 16016061
Avidin Biotin Blocking system	BioLegend	Cat# 927301
DAPI	Dojin	Cat# D523
MG132	Chemscene	Cat# CS-0471
N-Ethylmaleimide	Fujifilm	Cat# 058-02061
Cycloheximide (CHX)	Fujifilm	Cat# 037-20991
cOmplete Protease Inhibitor Cocktail	Roche	Cat# 11873580001
cOmplete, EDTA-free Protease Inhibitor Cocktail	Roche	Cat# 4693159001
PMSF	Sigma-Aldrich	Cat# P7627
NaF	Fujifilm	Cat# 192-01972
Na_3_VO_4_	Fujifilm	Cat# 198-09752
Bovine Serum Albumin	Sigma-Aldrich	Cat# A2153-50G
HBSS	Gibco	Cat# 24020-117
Type IV collagenase	Sigma-Aldrich	Cat# C4-28-100MG
DNase I (for tissue homogenize)	Sigma-Aldrich	Cat# 11284932001
Percoll	Cytiva	Cat# 17544502
Trizol reagent	Thermo Fisher Scientific	Cat# 10296010
DNase I (for RNA purification)	Takara bio	Cat# 2270A
RNase inhibitor	TOYOBO	Cat# SIN-201X5
3xFlag peptide	Sigma-Aldrich	Cat# F4799
anti-Myc magnetic beads	MBL	Cat# 3340
HA-Trap HA-Trap Magnetic Particles M-270	Proteintech	Cat# ATD-10
Dynabeads Protein G	Thermo Fisher Scientific	Cat# 10003D
Lympholyte	CEDARLANE	Cat# CL5115
7AAD	BioLegend	Cat# 420404
DMSO (Dimethyl sulfoxide)	Sigma-Aldrich	Cat# D2650
NEBNext dsDNA Fragmentase	New England Biolabs	Cat# M0348

**Critical commercial assays**		

KOD FX *Neo*	TOYOBO	Cat# KFX-201
NucleoBond Xtra Midi EF	MACHEREY-NAGEL	Cat# 740420
Neon™ Transfection System 10 μL Kit	Thermo Fisher Scientific	Cat# N1096
BD Cytofix/Cytoperm Kit	BD Pharmingen	Cat# 554714
DAB substrate kit	Vector lab	Cat# SK-4100
EzFastBlot	ATTO	Cat# AE-1465
EzBlock Chemi	ATTO	Cat# AE-1475
Can Get Signal solution1 & 2	TOYOBO	Cat# NKB-101
Western BLoT Rapid Detect v2.0	Takara	Cat# T7122A
Immobilon Western Chemiluminescent HRP substrate	Millipore	Cat# WBKLS0100
Trizol Reagent	Invitrogen	Cat# 15596026
DNaseI	TAKARA	Cat# 2270A
RNase Inhibitor Recombinant	TOYOBO	Cat# SIN-201X5
Glycogen	Invitrogen	Cat# AM9510
Nuclease free water	Invitrogen	Cat# AM9337
3M Sodium Acetate	Nakarai	Cat# 06893-24
ReverTra Ace qPCR RT Master Mix	TOYOBO	Cat# FSQ-201
THUNDERBIRD Next SYBRTM qPCR Mix	TOYOBO	Cat# QPX-201
Qubit RNA BR Assay Kit	Thermo Fisher Scientific	Cat# Q10211
RNA 6000 Nano Kit	Agilent Technologies	Cat# 5067-1511
NEBNext Poly(A) mRNA Magnetic Isolation Module	New England Biolabs	Cat. No. E7490
NEBNext Ultra II DNA library Prep kit for Illumina	New England Biolabs	Cat. No. E7645S
Qubit@ 2.0 Fluorometer	Thermo Scientific	Cat #Q32866

**Deposited data**		

RNA-seq of Liver and Colon from WT mice and *Ambra1* cKO mice	This paper	DDBJ: DRA017359
Exome analysis of Tumor from WT mice and *Ambra1* cKO mice	This paper	DDBJ: DRA017449

**Experimental models: Cell lines**		

OVA53	T. Sato et al.^42^	N/A
*Ambra1*^*flox/flox*^ MEF	This paper	
*Ambra1*^*flox/flox*^*Rosa-Cre-ERT2-Tg* MEF	This paper	N/A
*cA-H-Ras*^*G12V*^*Ambra1*^*flox/flox*^*Rosa-Cre-ERT2-Tg* MEF	This paper	N/A
*cA-H-Ras*^*G12V*^*Rosa-Cre-ERT2-Tg* MEF	This paper	N/A
Plat-E		

**Experimental models: Organisms/strains**		

Mouse: *Ambra1*^*flox/flox*^*Rosa-Cre-ERT2-Tg*	This paper	N/A

**Oligonucleotides**		

*Ambra1* exon2 KO check F 5′-GGTGCGACAGTGGCTCCTGA-3′	N/A	N/A
*Ambra1* exon2 KO check R 5′-AACTGCCTGATAGTCCACG-3′	N/A	N/A
*Ambra1* exon 4 F 5′-CCCTAGAGACCCT TTGAAGCACTGG-3′	N/A	N/A
*Ambra1* exon 4 R 5′-CCTGTGAACATT CCAGCTTGGTGC-3′	N/A	N/A
*Cre-ERT2* 1 5′- AAAGTCGCTCTGAGTTGTTAT-3′	N/A	N/A
*Cre-ERT2* 2 5′-GCGAAGAGTTTGTCCTCAACC-3′	N/A	N/A
*Cre-ERT2* 3 5′-GGAGCGGGAGAAATGGATATG-3′	N/A	N/A
*cA-H-Ras* F 5′-TCAGCAGCCTCCCTTCTGCC-3′	N/A	N/A
*cA-H-Ras* R 5′-GAAAACCAAGATCAAGACCA-3′	N/A	N/A
*cyclin d1* F for qPCR 5′- CATCCATGCGGAAAATCG -3′	N/A	N/A
*cyclin d1* R for qPCR 5′- CAGGCGGCTCTTCTTCAA -3′	N/A	N/A
*cyclin d2* F for qPCR 5′- GGCCAAGATCACCCACACT -3′	N/A	N/A
*cyclin d2* R for qPCR 5′- ATGCTGCTCTTGACGGAACT -3′	N/A	N/A
*cyclin d3* F for qPCR 5′- GGCATACTGGATGCTGGAG -3′	N/A	N/A
*cyclin d3* R for qPCR 5′- CCAGGTAGTTCATAGCCAGAGG -3′	N/A	N/A

**Recombinant DNA**		

human *AMBRA1* cDNA	DANAFORM	clone ID 5296472
pGCDNsam IRES hNGFR	Akatsuka et al.^12^	N/A
pGCDNsam AMBRA1-WT IRES hNGFR	Akatsuka et al.^12^	N/A
pGCDNsam AMBRA1-Mut IRES hNGFR	This paper	N/A
pGCDNsam AMBRA1-flag-myc IRES hNGFR	This paper	N/A
pAZG IRES NeoR	This paper	N/A
pAZG mCherry-Cdt1 IRES NeoR	This paper	N/A
pAZG mVenus-Geminin IRES NeoR	This paper	N/A
pAZG mcyclin D3-HA IRES PuromycinR	This paper	N/A
pAZG mcyclin CDK4-HA IRES PuromycinR	This paper	N/A
pAZG mcyclin CDK6-HA IRES PuromycinR	This paper	N/A
pCX mPB	Gift from Dr. Masato Ohtsuka	N/A

**Software and algorithms**		

FlowJo Version 10	Tree Star Inc.	RRID:SCR_008520
Fiji	NIH	RRID:SCR_002285
SPSS	IBM	RRID:SCR_002865
R v3.6.1	The R Foundation	RRID:SCR_001905
Trimmomatic (v0.39)	Bolger et al.^43^	*RRID*:SCR_011848
STAR (v2.7.3a)	Dobin et al.^44^	RRID:SCR_004463
RSEM (v1.3.1)	Li et al.^45^	RRID:SCR_000262
tximport v1.12.3	Soneson et al.^46^	RRID:SCR_016752
edgeR (v3.26)	Robinson et al.^47^	RRID: SCR_012802
TrimGalore (version 0.6.3)	Babraham Institute	RRID:SCR_011847
BWA-MEM (version 0.7.17)	Depristo et al.^48^ McKenna et al.^49^	RRID:SCR_022192
SAMtools (version 1.9)	Li et al.^50^	RRID:SCR_002105
Picard tools (version 2.20.6)	Broad Institute	RRID:SCR_006525
GATK (version 4.1.3.0)	Depristo et al.^48^ McKenna et al.^49^	RRID:SCR_001876
Strelka2 (version 2.9.10)	Kim et al.^51^	RRID:SCR_005109
Manta (version 1.6.0)	Chen et al.^52^	RRID:SCR_022997
Ensembl Variant Effect Predictor	McLaren et al.^53^	RRID:SCR_007931

### Experimental model and study participant details

#### Mice

The generation of *Ambra1*^*flox/flox*^ mice has been described previously.^19^ *Rosa-Cre-ERT2*-*Tg* mice^54^ were crossed with *Ambra1*^*flox/flox*^ to obtain *Ambra1*^*flox/flox*^*-Rosa-Cre-ERT2-Tg(+)* and *Ambra1*^*flox/flox*^*-Rosa-Cre-ERT2-Tg(−)* mice as littermate controls. For the conditional deletion of *Ambra1* exon 4, 100 μL of Tam dissolved in corn oil was subcutaneously injected at a final concentration of 20 mg/mL into 8-week-old mice once a day (three times). *cA-H-Ras* (conditionally active *H-Ras G12V*)*-Tg* mice were generated by microinjecting fertilized eggs with the *H-Ras G12V* gene, in which the first intron contained a spacer segment with a stop sequence and Poly(A) signal, flanked by a *loxP* sites. In the absence of Cre recombinase, the transcribed transgene was mis-spliced and not translated. In contrast, the active (G12V) H-RAS was translated by Cre recombination. *cA-H-Ras* mice were intercrossed with *Ambra1*^*flox/flox*^*-Rosa-Cre-ERT2-Tg(+)* mice. The daily food intake was measured individually for each subject prior to tamoxifen administration and again after 5 weeks. To detect the cells that entered the S phase *in vivo*, BrdU in phosphate-buffered saline (PBS) (10 mg/mL) was injected intraperitoneally (100 mg/kg) 24 h before the experiment. For the chemically-induced cancer model, mice were injected intraperitoneally with azoxymethane (10 mg/kg). For X-ray irradiation-induced tumors, the mice were X-ray-irradiated (8.5 Gy) using the MBR-1520R-3 apparatus (Hitachi, Japan) and transferred with 1–10 × 10^6^ BM cells from *Ambra1*^*flox/flox*^*-Rosa-Cre-ERT2-Tg(−)* mice two weeks before the administration of Tam. The extent of carcinogenesis was examined in surviving mice after 25 weeks. The mice were maintained under specific pathogen-free conditions at the animal facility of the Tokai University School of Medicine. All animal protocols were approved by the Institutional Animal Care and Use Committee of Tokai University.

#### Cell lines and cell culture conditions

OVA53 cells are murine thymic lymphoma-derived CD4^+^8^+^ immature T cells.^22^ Using CRISPR/Cas9, *Ambra1* cKO cells were obtained as previously reported.^12^ OVA53 cells were cultured in RPMI 1640 medium containing 10% fetal calf serum, 20 mM HEPES, 10 mM sodium pyruvate, 2 mM glutamate, 55 nM 2-mercaptoethanol (2-ME), penicillin (100 μg/mL), and streptomycin (100 μg/mL). To deliver the TCR-mediated signal, the cells were cultured with a hamster anti-mouse CD3ε antibody at a concentration of 250 ng/mL in plastic culture plates pre-coated with anti-hamster IgG. MEFs were prepared from the embryos of *Ambra1*^*flox/flox*^, *Ambra1*^*flox/flox*^ *Rosa-Cre-ERT2-Tg*, *cA-H-Ras*^*G12V*^ *Ambra1*^*flox/flox*^ *Rosa-Cre-ERT2-Tg*, and *cA-H-Ras*^*G12V*^ *Rosa-Cre-ERT2-Tg* mice on gestational day 12.5 MEFs were cultured in Dulbecco’s modified Eagle’s medium supplemented with 10% fetal calf serum, 2 mM glutamate, 55 nM 2-ME, 100 mg/mL penicillin, and 100 mg/mL streptomycin. The MEFs were expanded by passaging them three times, dispensed, frozen in liquid nitrogen using the CELLBANKER, and thawed for experimental purposes. To delete *Ambra1* exon 4 and express active Ras, MEFs were cultured in a 4-hydroxytamoxifen-containing medium. Plat-E cells were maintained in Dulbecco’s modified Eagle’s medium supplemented with 10% fetal calf serum, 2 mM glutamate, 55 nM 2-ME, 100 mg/mL penicillin, and 100 mg/mL streptomycin. All cells were maintained at 37°C in 5% CO_2_. Mycoplasma contamination testing was not performed for the cell lines used in this study.

### Method details

#### Reagents and antibodies

The key resources table lists all reagents, resources, and antibodies used in this study.

#### Plasmids and transfection

All plasmids used in the study are listed in the key resources table.

pGCDNsamIREShuKO was provided by Dr. M. Onodera.^55^ The huKO fragment was replaced with an hNGFR fragment, into which human *AMBRA1* cDNA (DNAFORM clone ID 5296472) was inserted. Recombinant retroviruses were produced using Plat-E cells and infected into OVA53 cells, as previously reported.^12^ mCherry-hCdt1 and mVenus-Geminin were amplified using PCR from CSII-EF-MCS mVenus-hGeminin and CSII-EF-MCS mCherry-hCdt1 were provided by Dr. A. Miyawaki.^56^ PCR products were inserted into a PiggyBac vector (pAZG and pAZG IRES NeoR). Murine *cyclind3*, *Cdk4*, and *Cdk6* cDNAs were amplified using PCR and inserted into a PiggyBac vector (pAZG IRES PuromycinR) with an HA-tag. According to the manufacturer’s instructions, the pAZG vectors were co-transfected with pCX mPB using the Neon transfection system (Invitrogen MPK5000, Thermo Fischer Scientific). The infected cells were collected using fluorescence-activated cell sorting (FACS) AriaIII (BD Biosciences) or selected in the presence of G418 (600 μg/mL) or puromycin (1 μg/mL). Unless otherwise indicated, the collected or selected cells were used for experiments without clonal isolation.

#### Immunoblot analysis

Total cell lysates were prepared using RIPA buffer (50 mM Tris-HCl pH 7.5, 150 mM NaCl, 1% NP-40, 1% sodium deoxycholate, and 0.1% sodium dodecyl sulfate [SDS]) with a protease inhibitor cocktail, 0.5 mM phenylmethylsulfonyl fluoride, 0.1 M NaF, and 0.1 M Na_3_VO_4_. Proteins were separated on an SDS-polyacrylamide gel and transferred onto a polyvinylidene fluoride membrane. The membranes were blocked with 3% BSA in tris-buffered saline containing 0.05% Tween (TBST) or EZBlock Chemi (ATTO) and incubated for 15h at 4°C with primary antibodies diluted in 3% BSA-TBST or Can Get Signal solution 1 (TOYOBO). After washing with TBST, the membranes were incubated with horseradish peroxidase (HRP)-conjugated secondary antibodies diluted in TBST or Can Get Signal solution 2 (Toyobo) or with HRP directly conjugated to the primary antibody using Western BLoT Rapid Detect v2.0 (Takara) for 1 h at 4°C. After washing with TBST, the target proteins were detected using the Immobilon Western Chemiluminescent HRP substrate (Millipore). Band intensities were measured using the *ImageJ* software.

#### Immunoprecipitation

Cells were lysed with immunoprecipitation (IP) lysis buffer (50 mM Tris-HCl pH 7.5, 150 mM NaCl, 10% glycerol, 0.1% Tween, and 1 mM EDTA) containing a protease inhibitor cocktail, 0.5 mM phenylmethylsulfonyl fluoride, 0.1 M NaF, 0.1 M Na_3_VO_4_. The protein concentration was measured using a DC protein assay (Bio-Rad). 1 mg of protein lysate was incubated with antibodies in IP lysis buffer and rotated at 4°C for 16 h. Precleared Dynabeads protein G (Thermo Fisher Scientific) were then added and rotated at 4°C for 2 h. The beads were washed three times with IP lysis buffer using a magnetic stand. Proteins were resuspended in 2 × SDS sample buffer and boiled for 5 min. After centrifugation, the supernatant was subjected to immunoblot analysis.

#### Histological analysis

The tissues were fixed with 4% paraformaldehyde and embedded in paraffin, and thin paraffin sections (2.25 μm) were stained with hematoxylin and eosin. For immunofluorescence and immunohistochemistry analyses, antigen retrieval was performed by heating (110°C) the sections in citrate buffer (pH 6.0). The sections were blocked with 5% normal goat serum and incubated with primary antibodies. After washing, the sections were incubated with Alexa 488-, Alexa 594-, or horseradish peroxidase-conjugated secondary antibodies. For immunohistochemical analysis, internal peroxidases were blocked following incubation with primary antibodies. The cell nuclei were stained with 4′,6-diamidino-2-phenylindole, or hematoxylin. Images were captured using an upright BX63 microscope (Olympus) or Axio Imager M2 (Carl Zeiss). Images were analyzed using the *ImageJ* software.

#### Preparation of hematopoietic cells from the liver and lungs

According to the manufacturer’s instructions, hematopoietic cells were harvested from the liver and lungs using a gentleMACS Octo Dissociator with Heaters system (Miltenyi Biotec). Briefly, after the perfusion of mice with PBS, the liver and lungs were chopped into small pieces in a gentle MACS C tube (Miltenyi Biotec). The pieces were dissociated with digestion buffer (10 mL Hank’s balanced salt solution [HBSS] supplemented with 0.01% type IV collagenase, 0.02% bovine serum albumin [BSA], 0.001% DNase I, and 1 mM CaCl_2_ for the lungs; 5 mL HBSS supplemented with 0.1% type IV collagenase and 0.01% DNase I for the liver) and processed in a MACS Octo Dissociator. Ice-cold 2% fetal calf serum/PBS was added to the digested samples and filtered through a nylon mesh. After washing with PBS, the cell pellet was resuspended in 5 mL of 30% Percoll in HBSS, overlaid onto 70% Percoll in HBSS, and centrifuged at 800 × *g* at 25°C for 25 min. The cells were collected from the interface, washed with PBS, and analyzed using FACS.

#### Flow cytometry analysis

OVA53 cells and murine hematopoietic cells from the peripheral blood, BM, lymph nodes, lungs, and liver were stained with fluorescence-labeled antibodies. The cells were cultured with BrdU (10 μM) for 30 min for cell cycle analysis. Cells were fixed and permeabilized using Cytofix/Cytoperm and stained with an allophycocyanin-conjugated anti-BrdU antibody. The stained and unstained control cells were analyzed using a FACSverse or LSRFortessa (BD Biosciences).

#### RNA extraction and qPCR analysis

Total RNA was extracted using TRIzol Reagent (Invitrogen). cDNA was synthesized with the ReverTra Ace qPCR RT Master Mix (TOYOBO). SYBR Green-based qPCR was performed using a QuantStudio 5 Real-Time PCR system with the THUNDERBIRD Next SYBRTM qPCR Mix (TOYOBO). Gene expression values were normalized to *Ubc* (*Ubiquitin C*) gene. The sequences of specific primers are listed in the key resources table.

#### RNA sequencing

RNA was extracted using the Trizol reagent. The quantity and quality of the extracted RNA samples were evaluated using the Qubit RNA BR Assay Kit and the Agilent Bioanalyzer 2100 RNA 6000 Nano Kit, respectively. One microgram of total RNA was used for oligo-dT-based mRNA isolation using the NEBNext Poly(A) mRNA Magnetic Isolation Module (New England Biolabs, Ipswich, MA, USA). Libraries were prepared using the NEBNext Ultra Directional RNA Library Prep Kit for Illumina. Libraries were sequenced using an Illumina NovaSeq 6000 platform in rapid run mode with a 2 × 100-bp paired-end module (Illumina). Data availability: The accession number for the RNA sequence is DDBJ DRA017359.

#### Analyses of differentially expressed genes

Low-quality reads and adapter sequences were removed using Trimmomatic (v0.39).^43^ The trimmed reads were then mapped to the GRCm38 reference genome using STAR (v2.7.3a),^44^ and gene expression was estimated using RSEM (v1.3.1)^45^ with the GENCODE M25 gene annotation. The estimated gene expression values were subjected to differential gene expression analysis using tximport v1.12.3.^46^ The exact test in edgeR (v3.26)^47^ was performed to determine differential expression.

#### Whole-exome sequencing for tumors in mice

##### Experimental procedure

SNVs and InDels were identified in each pair of tumors, and matched blood samples were collected from the same mouse. For each sample, 200 ng of DNA was fragmented using NEBNext dsDNA Fragmentase. Sequencing libraries were constructed using the NEBNext Ultra II DNA Library Prep Kit for Illumina (New England BioLabs). Libraries were hybridized to probes of SeqCap EZ Mouse Exome Design (Roche Diagnostics, Basel, Switzerland). The quantity and size distribution of the captured libraries were assessed using a Qubit 2.0 Fluorometer (Thermo Fisher Scientific) and a Bioanalyzer 2100 (Agilent Technologies, Santa Clara, CA, USA), respectively. Libraries were sequenced using the Illumina HiSeq 2500 platform with a 2 × 150-bp paired-end module (Illumina).

##### Preprocessing of sequence data

Bioinformatics analyses were performed based on our previous studies.^36^^,^^40^ Illumina adaptor sequences were trimmed for quality control using TrimGalore (version 0.6.3) (https://www.bioinformatics.babraham.ac.uk/projects/trim_galore/). Low-quality sequences were excluded or trimmed using the Trimmomatic software (version 0.39).^43^ The filtered sequence reads were aligned to the mouse reference genome (GRCm38) using BWA-MEM (version 0.7.17).^48^^,^^49^ Sequence alignment map (SAM) files were sorted and converted to a binary alignment map file format using SAMtools (version 1.9).^50^ To remove the PCR duplicates, binary alignment map files were processed using Picard tools (version 2.20.6) (https://broadinstitute.github.io/picard/). Base quality recalibration was performed using GATK (version 4.1.3.0).^48^^,^^49^ The average depth and coverage of the target regions were calculated using SAM tools.

##### Detection of somatic mutations

SNVs and InDels were identified in each pair of tumors, and matched blood samples were collected from the same mouse using Strelka2 (version 2.9.10).^51^ To detect somatic InDels, we used information on candidate InDel sites provided by Manta (version 1.6.0).^52^ Somatic mutations satisfying the following criteria were used: i) empirical variant score for the somatic mutation provided by Strelka2 greater than 13.0103 (−10 × log_10_ 0.05); ii) sequencing depth at the mutation site in tumor samples greater than or equal to 20; iii) number of sequence reads supporting the mutant allele in tumor samples greater than or equal to eight; iv) number of sequences reads supporting the mutant allele in matched blood samples less than two; and v) mutant allele frequency in matched blood samples less than 0.05.

##### Functional annotations of the identified mutations

Functional annotations for protein-coding and transcription-related effects of the identified somatic mutations were performed using Ensembl Variant Effect Predictor.^53^ Data availability: The accession number for the exome sequence is DDBJ DRAS000547.

#### Two-step affinity purification of AMBRA1 complexes

*Ambra1*-deficient OVA53 cells were infected with Myc, and FLAG-tagged AMBRA1 was inserted into the pGCDN-hNGFR retroviral vector. After infection, hNGFR^+^ cells were sorted using FACS Aria III (BD Biosciences). The sorted cells were analyzed using a previously reported method.^57^ Briefly, hNGFR^+^ OVA53 cells were solubilized with immunoprecipitation lysis buffer supplemented with a protease inhibitor cocktail (4693159001, Roche) and 1 mM dithiothreitol, lysed on ice for 30 min with gentle shaking, and sonicated on a VP-55 sonicator (TAITEC) for three cycles (output control “7” for 20 s followed by 20 s rest). Immunoprecipitation with anti-Flag M2 agarose (Sigma-Aldrich) was performed overnight at 4°C. The complexes were eluted from agarose gel using 3×Flag peptide, and the eluted AMBRA1 complexes were subjected to a second immunoprecipitation step with anti-Myc magnetic beads. Immune complexes were eluted from the beads with Myc peptide and separated using SDS-PAGE. The bands were excised from the gel and subjected to mass spectrometric analysis to identify the corresponding proteins using the Advance UHPLC (Bruker) and Orbitrap Velos Pro Mass Spectrometer (Thermo Fisher Scientific).

### Quantification and statistical analysis

Survival curves were plotted using Kaplan-Meier estimates with the log rank test to determine significant differences. The frequency of tumor incidence was estimated using the chi-square test. The number of cell nuclei was analyzed using the Steel-Dwass test for post hoc nonparametric multiple comparisons. For the other *in vivo* experiments, the data were analyzed using the Mann-Whitney U test. Data from the *in vitro* experiments were analyzed using a two-tailed *t-*test, one-way ANOVA, or two-way ANOVA with Tukey’s post hoc test.

## Supplemental information

Document S1. Figures S1–S11 and Tables S4

Table S1. List of AMBRA1 mutations found in tumors registered in the cBioPortal related to Figure 1 *AMBRA1* mutations found in tumors registered in cBioPortal were extracted. Two mutations (mutation1: A89G:Q30R and mutation2: G3585C:R1195S) identified in patients with Cowden syndrome were not among the *AMBRA1* mutations registered in the cBioPortal.

Table S2. Two-step affinity purification of AMBRA1 complexes from OVA53 cells related to Figure S5 The total numbers of binding molecules (A) and ubiquitin-related molecules (B) are listed. Mascot score is the probability that the observed match is a random event (Mascot score 100 means absolute probability 1 × 10−10).

Table S3. Transcriptome analysis of colon and liver of Ambra1 cKO mouse related to Figure S10 Differentially expressed genes (DEGs) were extracted using edgeR, with FDR<0.05, |Log2FC|>1, and TPM>1 (in WT or Ambra1 KO), based on five biologically independent replicates for colon (A) and liver (b). Transcriptome analysis revealed a relatively small number of differentially expressed genes, particularly in the colon. Ambra1 functions primarily at the post-transcriptional level. (C) TPM of cyclin D1, cyclinD2, and cyclin D3 of colon and liver in control mice (WT) and Ambra1 cKO mice (KO).

Data S1. Supplemental data Western Blot original data

## References

[1] Marescal O., Cheeseman I.M. (2020). Cellular Mechanisms and Regulation of Quiescence. Dev. Cell.

[2] Pilarski R. (2019). PTEN Hamartoma Tumor Syndrome: A Clinical Overview. Cancers (Basel).

[3] Hollander M.C., Blumenthal G.M., Dennis P.A. (2011). PTEN loss in the continuum of common cancers, rare syndromes and mouse models. Nat. Rev. Cancer.

[4] Milella M., Falcone I., Conciatori F., Cesta Incani U., Del Curatolo A., Inzerilli N., Nuzzo C.M.A., Vaccaro V., Vari S., Cognetti F., Ciuffreda L. (2015). PTEN: Multiple Functions in Human Malignant Tumors. Front. Oncol..

[5] Liaw D., Marsh D.J., Li J., Dahia P.L., Wang S.I., Zheng Z., Bose S., Call K.M., Tsou H.C., Peacocke M. (1997). Germline mutations of the PTEN gene in Cowden disease, an inherited breast and thyroid cancer syndrome. Nat. Genet..

[6] Revathidevi S., Hosomichi K., Natsume T., Nakaoka H., Fujito N.T., Akatsuka H., Sato T., Munirajan A.K., Inoue I. (2022). AMBRA1 p.Gln30Arg Mutation, Identified in a Cowden Syndrome Family, Exhibits Hyperproliferative Potential in hTERT-RPE1 Cells. Int. J. Mol. Sci..

[7] Fimia G.M., Stoykova A., Romagnoli A., Giunta L., Di Bartolomeo S., Nardacci R., Corazzari M., Fuoco C., Ucar A., Schwartz P. (2007). Ambra1 regulates autophagy and development of the nervous system. Nature.

[8] Nazio F., Strappazzon F., Antonioli M., Bielli P., Cianfanelli V., Bordi M., Gretzmeier C., Dengjel J., Piacentini M., Fimia G.M., Cecconi F. (2013). mTOR inhibits autophagy by controlling ULK1 ubiquitylation, self-association and function through AMBRA1 and TRAF6. Nat. Cell Biol..

[9] Antonioli M., Albiero F., Nazio F., Vescovo T., Perdomo A.B., Corazzari M., Marsella C., Piselli P., Gretzmeier C., Dengjel J. (2014). AMBRA1 Interplay with Cullin E3 Ubiquitin Ligases Regulates Autophagy Dynamics. Dev. Cell.

[10] Cianfanelli V., Fuoco C., Lorente M., Salazar M., Quondamatteo F., Gherardini P.F., De Zio D., Nazio F., Antonioli M., D’Orazio M. (2015). AMBRA1 links autophagy to cell proliferation and tumorigenesis by promoting c-Myc dephosphorylation and degradation. Nat. Cell Biol..

[11] Cianfanelli V., De Zio D., Di Bartolomeo S., Nazio F., Strappazzon F., Cecconi F. (2015). Ambra1 at a glance. J. Cell Sci..

[12] Akatsuka H., Kuga S., Masuhara K., Davaadorj O., Okada C., Iida Y., Okada Y., Fukunishi N., Suzuki T., Hosomichi K. (2017). AMBRA1 is involved in T cell receptor-mediated metabolic reprogramming through an ATG7-independent pathway. Biochem. Biophys. Res. Commun..

[13] Simoneschi D., Rona G., Zhou N., Jeong Y.T., Jiang S., Milletti G., Arbini A.A., O’Sullivan A., Wang A.A., Nithikasem S. (2021). CRL4AMBRA1 is a master regulator of D-type cyclins. Nature.

[14] Chaikovsky A.C., Li C., Jeng E.E., Loebell S., Lee M.C., Murray C.W., Cheng R., Demeter J., Swaney D.L., Chen S.-H. (2021). The AMBRA1 E3 ligase adaptor regulates the stability of cyclin D. Nature.

[15] Maiani E., Milletti G., Nazio F., Holdgaard S.G., Bartkova J., Rizza S., Cianfanelli V., Lorente M., Simoneschi D., Di Marco M. (2021). AMBRA1 regulates cyclin D to guard S-phase entry and genomic integrity. Nature.

[16] Chung M., Liu C., Yang H.W., Köberlin M.S., Cappell S.D., Meyer T. (2019). Transient Hysteresis in CDK4/6 Activity Underlies Passage of the Restriction Point in G1. Mol. Cell.

[17] Min M., Rong Y., Tian C., Spencer S.L. (2020). Temporal integration of mitogen history in mother cells controls proliferation of daughter cells. Science.

[18] Kaulich M., Link V.M., Lapek J.D., Lee Y.J., Glass C.K., Gonzalez D.J., Dowdy S.F. (2021). A Cdk4/6-dependent phosphorylation gradient regulates the early to late G1 phase transition. Sci. Rep..

[19] Quadros R.M., Miura H., Harms D.W., Akatsuka H., Sato T., Aida T., Redder R., Richardson G.P., Inagaki Y., Sakai D. (2017). Easi-CRISPR: A robust method for one-step generation of mice carrying conditional and insertion alleles using long ssDNA donors and CRISPR ribonucleoproteins. Genome Biol..

[20] Masuhara K., Akatsuka H., Tokusanai M., Li C., Iida Y., Okada Y., Suzuki T., Ohtsuka M., Inoue I., Kimura M. (2021). AMBRA1 controls antigen-driven activation and proliferation of naive T cells. Int. Immunol..

[21] Miura H., Quadros R.M., Gurumurthy C.B., Ohtsuka M. (2018). Easi-CRISPR for creating knock-in and conditional knockout mouse models using long ssDNA donors. Nat. Protoc..

[22] Sato T., Chiba T., Ohno S.I., Sato C., Sugoh T., Miyashita K., Akatsuka H., Hozumi K., Okada Y., Iida Y. (2012). Reciprocal control of G1-phase progression is required for Th-POK/Runx3-mediated CD4/8 thymocyte cell fate decision. J. Immunol..

[23] Zhao Y.M., Zhou Q., Xu Y., Lai X.Y., Huang H. (2008). Antiproliferative effect of rapamycin on human T-cell leukemia cell line Jurkat by cell cycle arrest and telomerase inhibition. Acta Pharmacol. Sin..

[24] Bronte V., Brandau S., Chen S.-H., Colombo M.P., Frey A.B., Greten T.F., Mandruzzato S., Murray P.J., Ochoa A., Ostrand-Rosenberg S. (2016). Recommendations for myeloid-derived suppressor cell nomenclature and characterization standards. Nat. Commun..

[25] Furman D., Campisi J., Verdin E., Carrera-Bastos P., Targ S., Franceschi C., Ferrucci L., Gilroy D.W., Fasano A., Miller G.W. (2019). Chronic inflammation in the etiology of disease across the life span. Nat. Med..

[26] Galdiero M.R., Marone G., Mantovani A. (2018). Cancer Inflammation and Cytokines. Cold Spring Harb. Perspect. Biol..

[27] Veglia F., Perego M., Gabrilovich D. (2018). Myeloid-derived suppressor cells coming of age. Nat. Immunol..

[28] Snider A.J., Bialkowska A.B., Ghaleb A.M., Yang V.W., Obeid L.M., Hannun Y.A. (2016). Murine Model for Colitis-Associated Cancer of the Colon. Methods Mol. Biol..

[29] Liu M., Wang Y., Teng F., Mai X., Wang X., Su M.Y., Stjepanovic G. (2023). Structure of the DDB1-AMBRA1 E3 ligase receptor complex linked to cell cycle regulation. Nat. Commun..

[30] Beumer J., Clevers H. (2021). Cell fate specification and differentiation in the adult mammalian intestine. Nat. Rev. Mol. Cell Biol..

[31] Ritsma L., Ellenbroek S.I.J., Zomer A., Snippert H.J., De Sauvage F.J., Simons B.D., Clevers H., Van Rheenen J. (2014). Intestinal crypt homeostasis revealed at single-stem-cell level by in vivo live imaging. Nature.

[32] Buczacki S.J.A., Zecchini H.I., Nicholson A.M., Russell R., Vermeulen L., Kemp R., Winton D.J. (2013). Intestinal label-retaining cells are secretory precursors expressing Lgr5. Nature.

[33] Mortensen M., Soilleux E.J., Djordjevic G., Tripp R., Lutteropp M., Sadighi-Akha E., Stranks A.J., Glanville J., Knight S., Jacobsen S.E.W. (2011). The autophagy protein Atg7 is essential for hematopoietic stem cell maintenance. J. Exp. Med..

[34] Cianfanelli V., Nazio F., Cecconi F. (2015). Connecting autophagy: AMBRA1 and its network of regulation. Mol. Cell. Oncol..

[35] Rona G., Miwatani-Minter B., Zhang Q., Goldberg H.V., Kerzhnerman M.A., Howard J.B., Simoneschi D., Lane E., Hobbs J.W., Sassani E. (2024). CDK-independent role of D-type cyclins in regulating DNA mismatch repair. Mol. Cell.

[36] Suda K., Nakaoka H., Yoshihara K., Ishiguro T., Tamura R., Mori Y., Yamawaki K., Adachi S., Takahashi T., Kase H. (2018). Clonal Expansion and Diversification of Cancer-Associated Mutations in Endometriosis and Normal Endometrium. Cell Rep..

[37] Martincorena I., Fowler J.C., Wabik A., Lawson A.R.J., Abascal F., Hall M.W.J., Cagan A., Murai K., Mahbubani K., Stratton M.R. (2018). Somatic mutant clones colonize the human esophagus with age. Science.

[38] Yokoyama A., Kakiuchi N., Yoshizato T., Nannya Y., Suzuki H., Takeuchi Y., Shiozawa Y., Sato Y., Aoki K., Kim S.K. (2019). Age-related remodelling of oesophageal epithelia by mutated cancer drivers. Nature.

[39] Anglesio M.S., Papadopoulos N., Ayhan A., Nazeran T.M., Noë M., Horlings H.M., Lum A., Jones S., Senz J., Seckin T. (2017). Cancer-Associated Mutations in Endometriosis without Cancer. N. Engl. J. Med..

[40] Yamaguchi M., Nakaoka H., Suda K., Yoshihara K., Ishiguro T., Yachida N., Saito K., Ueda H., Sugino K., Mori Y. (2022). Spatiotemporal dynamics of clonal selection and diversification in normal endometrial epithelium. Nat. Commun..

[41] Gottlieb S., Shang W., Ye D., Kubo S., Jiang P.D., Shafer S., Xu L., Zheng L., Park A.Y., Song J. (2024). AMBRA1 controls the translation of immune-specific genes in T lymphocytes. Proc. Natl. Acad. Sci. USA.

[42] Sato T., Ohno S.I., Hayashi T., Sato C., Kohu K., Satake M., Habu S. (2005). Dual functions of Runx proteins for reactivating CD8 and silencing CD4 at the commitment process into CD8 thymocytes. Immunity.

[43] Bolger A.M., Lohse M., Usadel B. (2014). Trimmomatic: a flexible trimmer for Illumina sequence data. Bioinformatics.

[44] Dobin A., Davis C.A., Schlesinger F., Drenkow J., Zaleski C., Jha S., Batut P., Chaisson M., Gingeras T.R. (2013). STAR: ultrafast universal RNA-seq aligner. Bioinformatics.

[45] Li B., Dewey C.N. (2011). RSEM: accurate transcript quantification from RNA-Seq data with or without a reference genome. BMC Bioinf..

[46] Soneson C., Love M.I., Robinson M.D. (2015). Differential analyses for RNA-seq: Transcript-level estimates improve gene-level inferences. F1000Res..

[47] Robinson M.D., McCarthy D.J., Smyth G.K. (2010). edgeR: a Bioconductor package for differential expression analysis of digital gene expression data. Bioinformatics.

[48] Depristo M.A., Banks E., Poplin R., Garimella K.V., Maguire J.R., Hartl C., Philippakis A.A., Del Angel G., Rivas M.A., Hanna M. (2011). A framework for variation discovery and genotyping using next-generation DNA sequencing data. Nat. Genet..

[49] McKenna A., Hanna M., Banks E., Sivachenko A., Cibulskis K., Kernytsky A., Garimella K., Altshuler D., Gabriel S., Daly M., DePristo M.A. (2010). The Genome Analysis Toolkit: a MapReduce framework for analyzing next-generation DNA sequencing data. Genome Res..

[50] Li H., Handsaker B., Wysoker A., Fennell T., Ruan J., Homer N., Marth G., Abecasis G., Durbin R., 1000 Genome Project Data Processing Subgroup (2009). The Sequence Alignment/Map format and SAMtools. Bioinformatics.

[51] Kim S., Scheffler K., Halpern A.L., Bekritsky M.A., Noh E., Källberg M., Chen X., Kim Y., Beyter D., Krusche P., Saunders C.T. (2018). Strelka2: fast and accurate calling of germline and somatic variants. Nat. Methods.

[52] Chen X., Schulz-Trieglaff O., Shaw R., Barnes B., Schlesinger F., Källberg M., Cox A.J., Kruglyak S., Saunders C.T. (2016). Manta: rapid detection of structural variants and indels for germline and cancer sequencing applications. Bioinformatics.

[53] McLaren W., Gil L., Hunt S.E., Riat H.S., Ritchie G.R.S., Thormann A., Flicek P., Cunningham F. (2016). The Ensembl Variant Effect Predictor. Genome Biol..

[54] Seibler J., Zevnik B., Küter-Luks B., Andreas S., Kern H., Hennek T., Rode A., Heimann C., Faust N., Kauselmann G. (2003). Rapid generation of inducible mouse mutants. Nucleic Acids Res..

[55] Sanuki S., Hamanaka S., Kaneko S., Otsu M., Karasawa S., Miyawaki A., Nakauchi H., Nagasawa T., Onodera M. (2008). A new red fluorescent protein that allows efficient marking of murine hematopoietic stem cells. J. Gene Med..

[56] Sakaue-Sawano A., Yo M., Komatsu N., Hiratsuka T., Kogure T., Hoshida T., Goshima N., Matsuda M., Miyoshi H., Miyawaki A. (2017). Genetically Encoded Tools for Optical Dissection of the Mammalian Cell Cycle. Mol. Cell.

[57] Hosokawa H., Romero-Wolf M., Yang Q., Motomura Y., Levanon D., Groner Y., Moro K., Tanaka T., Rothenberg E.V. (2020). Cell type-specific actions of Bcl11b in early T-lineage and group 2 innate lymphoid cells. J. Exp. Med..

