# Supplementary material for: Molecular and genetic evidence for the role of AMBRA1 in suppressing S-phase entry and tumorigenesis
Source: iScience. 2025 Jul 5;28(8):113054. doi: 10.1016/j.isci.2025.113054 (PMC12304916; doi:10.1016/j.isci.2025.113054)
Supplement: Document S1. Figures S1–S11 and Tables S4 [file mmc1.pdf]

iScience, Volume *28*

Supplemental information

Molecular and genetic evidence for the role of AMBRA1 in suppressing S-phase entry and tumorigenesis

Hisako Akatsuka, Tomohiro Kashikawa, Kaori Masuhara, Mizuki Tokusanai, Chenyang Li, Yumi Iida, Chisa Okada-Yamaguchi, Yoshinori Okada, Masayuki Tanaka, Takahiro Suzuki, Norio Yamamoto, Katsuto Hozumi, Tomoaki Tanaka, Hirofumi Nakaoka, Kazuyoshi Hosomichi, Yu Hamaguchi, Michiaki Hamada, Yoshiki Shiraishi, Akihide Kamiya, Yoshihiko Nakamura, Kaito Harada, Abd Aziz Ibrahim, Takashi Yahata, Masato Ohtsuka, Naoya Nakamura, Hiroyuki Hosokawa, Minoru Kimura, Ituro Inoue, and Takehito Sato

A

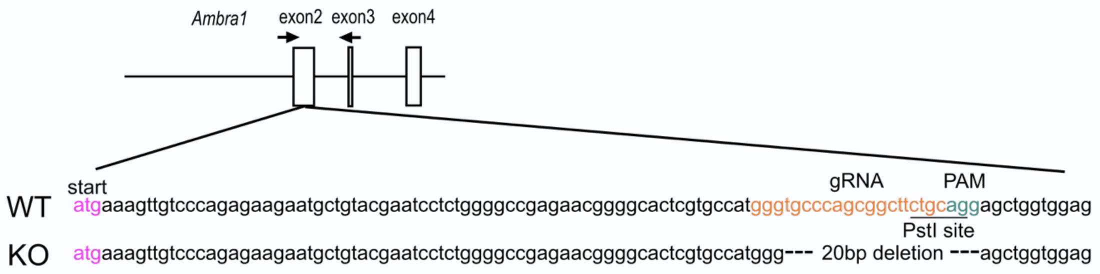

B

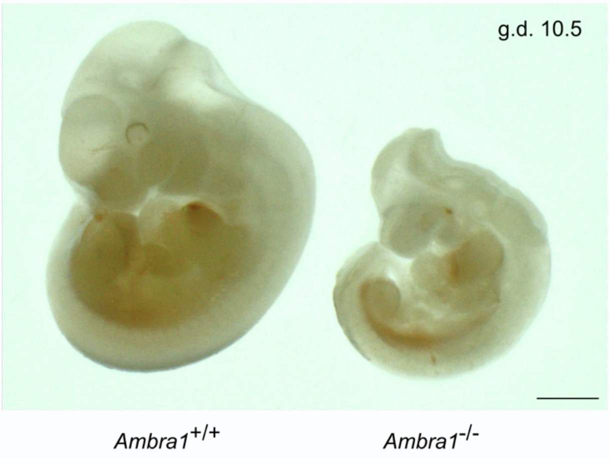

C

	+/+	+/-	-/-
e10.5	8 (29%)	14 (50%)	6 (21%)
e12.5	11 (40%)	15 (56%)	1 (4%)

Figure S1 *Ambra1* deficient mice have dysfunctional development, related to Figure 1.

(A) Using a CRISPR/Cas9 system, exon 2 of *Ambra1* in mouse eggs was disrupted. Mouse genotypes were determined by PCR using primers indicated by arrows, and the PCR products were digested with PstI. Sequencing of *Ambra1* exon2 without PstI sites revealed a 20-bp deletion allele.

(B) Image of embryos at gestational day 10.5. Scale bars, 1 mm.

(C) The table showing the numbers and percentage of fetuses with *Ambra1*+/+, +/-, and -/- genotypes. Heterogeneous mice (*Ambra1*+/-), which were healthy and fertile, were crossed to obtain fetuses with *Ambra1*+/+, +/-, and -/- at gestational days 10.5 and 12.5. As previously reported in *Ambra1*$^{gt/gt}$ mice, the development of *Ambra1*-/- mice was delayed, and they died by gestational day 14.5.

Akatsuka et al. Figure S2

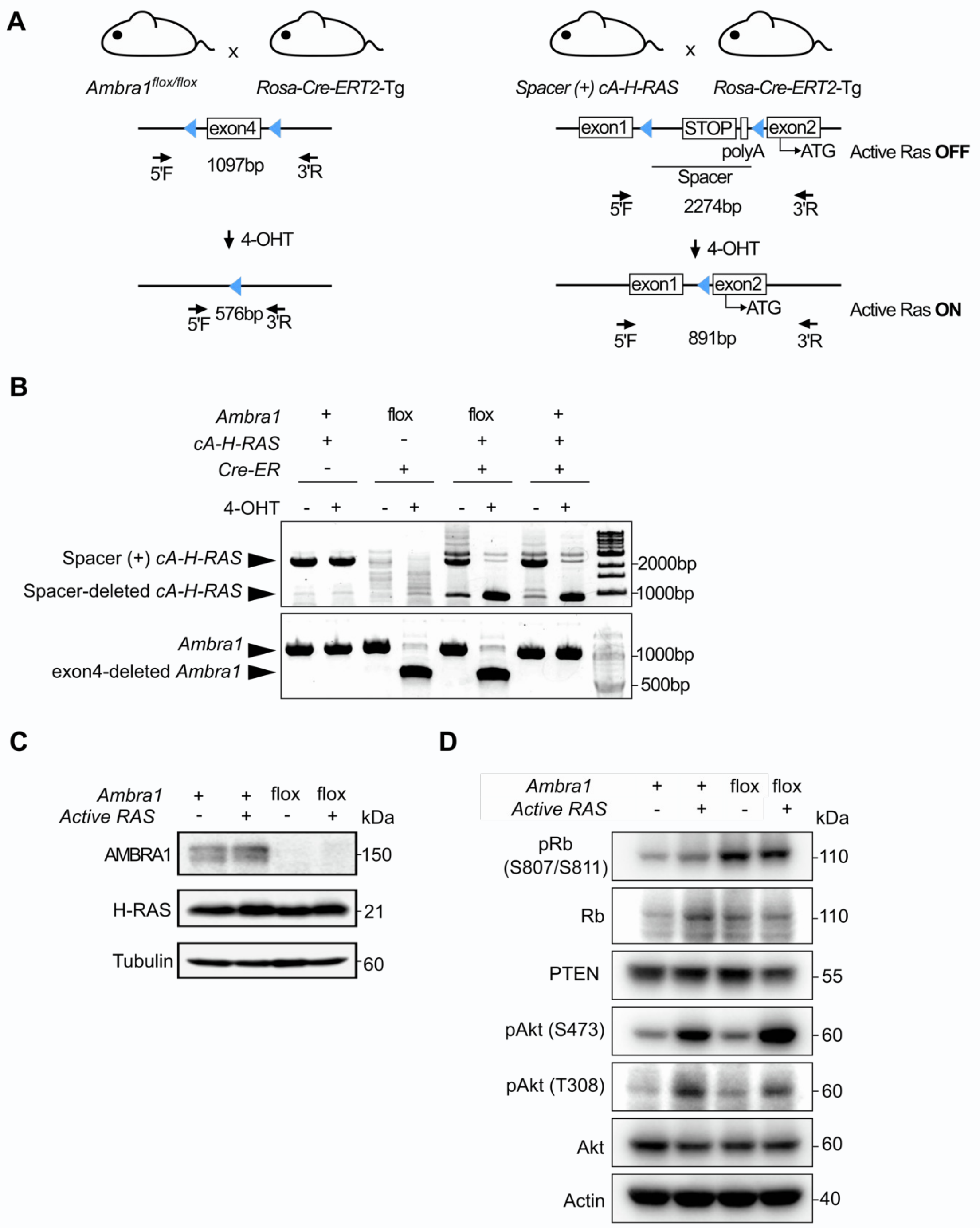

Figure S2 Establishment of conditional AMBRA1-deficient and active RAS-expressing mouse embryonic fibroblasts (MEFs), related to Figure 1.

(A) Fibroblasts obtained from embryos of the indicated genotypes. 4-Hydroxytamoxifen (4-OHT) treatment induced the deletion of *Ambra1* exon 4 in *Ambra1*f$^{flox/flox}$ or the stop sequence in spacer (+) *cA-H-RAS* when MEFs possessed the *Rosa-Cre-ERT2* transgene (Cre-ER+). PCR primers (5'F and 3'R) used to amplify deleted and undeleted segments are indicated by arrows. (B) MEFs were treated with or without 4-OHT for 5 days, and genomic DNA was extracted for PCR analysis. The transgenic conditional-active H-Ras G12V (cA-H-Ras, upper) and *Ambra1* exon 4 loci (lower) were amplified. Cre-mediated excision of the spacer sequence (spacer-deleted cA-H-Ras) enabled normal splicing and the expression of active H-RAS. (C) Immunoblot analysis of AMBRA1 and H-RAS expression in MEFs treated with 4-OHT from *Ambra1*$^{+/+}$ *cA-H-Ras* (-) *Rosa-Cre-ERT2* (+), *Ambra1*$^{+/+}$ *cA-H-Ras* (+) *Rosa-Cre-ERT2* (+), *Ambra1*f$^{flox/flox}$ *cA-H-Ras* (-) *Rosa-Cre-ERT2* (+), and *Ambra1*f$^{flox/flox}$ *cA-H-Ras* (+) *Rosa-Cre-ERT2* (+) mice. Tubulin was used as a loading control. (D) Immunoblot analysis of phospho-Rb (Ser807 / Ser811), Rb, phospho-Akt (Ser473 or Thr308), Akt, and Actin expression in MEFs from (C). Actin was used as a loading control.

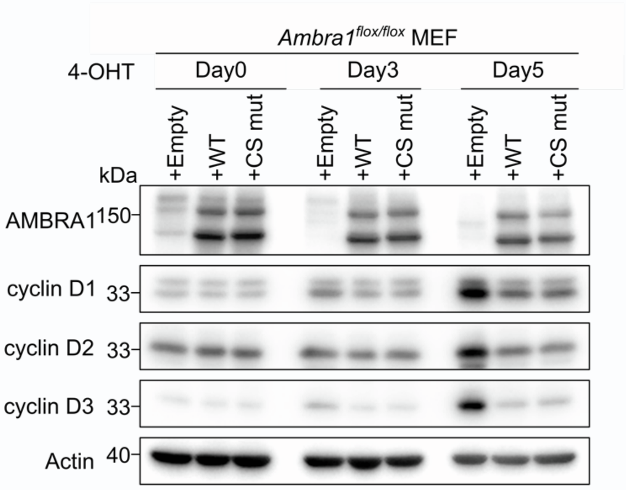

Figure S3 AMBRA1 suppresses the expression of D-type cyclins in MEF, related to Figure 1. *Ambra1*$^{flox/flox}$ MEFs were transfected with empty, AMBRA1 wild type- or AMBRA1 CS mutant- expressing vectors. After the addition of 4-OHT to the culture to deplete endogenous *Ambra1*, cells were harvested and lysed at day0, 3, 5, and the lysates were subjected to immunoblot analysis.

Akatsuka et al. Figure S4

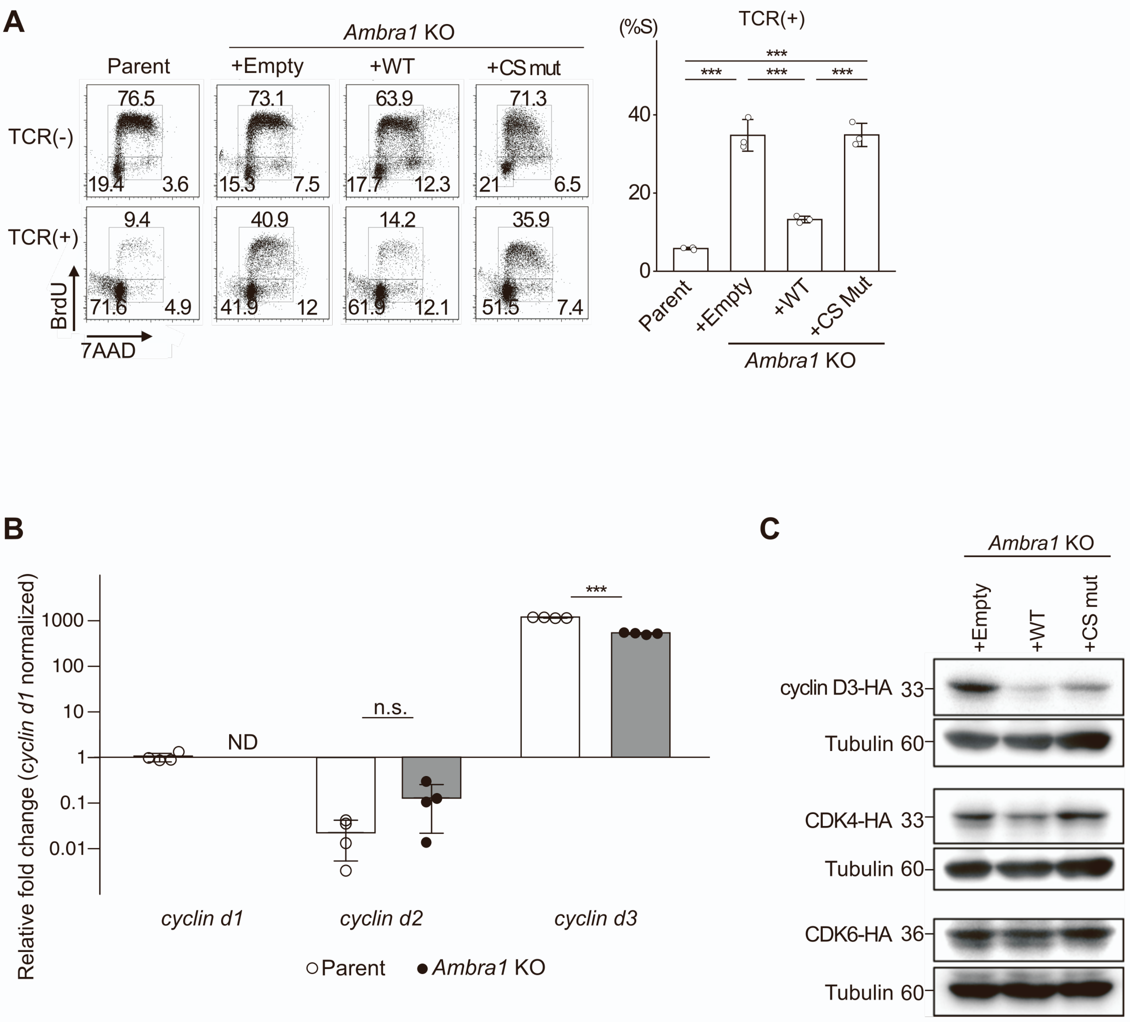

Figure S4 *Ambra1* deficient cells and mutant AMBRA1 expressing cells show the abnormal cell cycle control, related to Figure 2.

(A) Cells were pulsed with BrdU for 30 min. The percentages of BrdU-positive S-phase cells 48 h after TCR/CD3 crosslinking are indicated and shown as bar graphs. Mean ± SD of three cultures is shown. More than three independent experiments showed similar results. (***$p < 0.005$ in one-way ANOVA followed by Tukey's post hoc test).

(B) mRNA expression of *cyclin d1, cyclin d2, cyclin d3* in parental OVA53 cells and *Ambra1*-deficient OVA53 cells.

(C) *Ambra1*-deficient OVA53 cells expressing the empty vector (*Ambra1* KO +Empty), AMBRA1 WT (*Ambra1* KO +WT), and patient-type AMBRA1 mutant (*Ambra1* KO +CS Mut) were transfected with expression vectors carrying HA-tagged cyclin D3, CDK4, and CDK6. Immunoblotting was performed using antibodies against cyclin D3, CDK4, CDK6, and Tubulin.

A

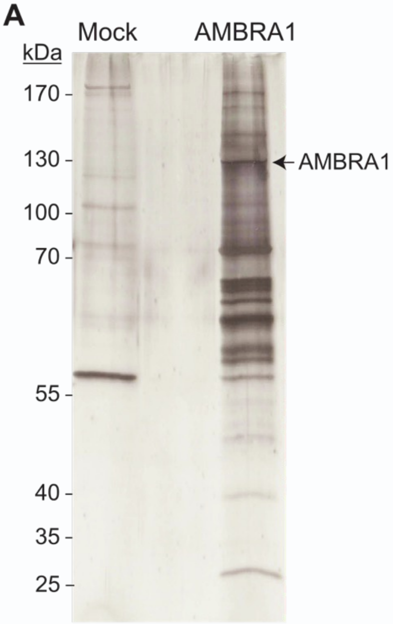

B

GO term	Fold enrichment	*P*-value	*FDR*
Mitotic nuclear division	3.90	6.87E-19	1.34E-15
Translation	3.29	9.18E-19	1.34E-15
Cell cycle	2.67	4.31E-17	4.19E-14
Cell division	3.23	8.01E-17	5.84E-14
Protein transport	2.58	5.84E-15	3.41E-12
Uniquitin-dependent protein catabolic process	4.59	7.06E-15	3.43E-12

C

KEGG pathway	Fold enrichment	*P*-value	*FDR*
Proteasome	9.33	4.66E-21	9.65E-19
RNA transport	3.61	8.14E-13	8.43E-11
Ribosome	3.82	2.17E-12	1.50E-10
DNA replication	7.28	1.47E-10	7.62E-09
Cell cycle	3.75	3.12E-10	1.29E-08
Ubiquitin mediated proteolysis	3.38	2.40E-09	8.29E-08

Figure S5 AMBRA1 interact with various proteins including Cell cycle and Ubiquitin related proteins, related to Figure 2.

(A) Extracts from Myc-Flag-AMBRA1-expressing OVA53 cells were subjected to two-step affinity purification, followed by SDS polyacrylamide gel electrophoresis (PAGE) and silver staining. The bands were excised from the gel and subjected to mass spectrometric analysis to identify the corresponding proteins using Advance UHPLC (Bruker) and Orbitrap Velos Pro Mass Spectrometer (Thermo Fisher Scientific) (Table S2).

(B) GO term analysis in Table S2 shows the proteins detected by liquid chromatography/mass spectrometry.

(C) KEGG pathway analysis of Table S2 showed LC/MS detected proteins.

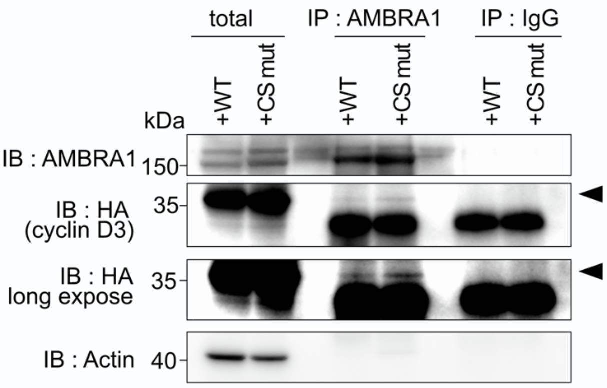

Figure S6 Binding to cyclin D3 was not reduced in AMBRA1 CS mutant, related to Figure 3.
Ambra1-disrupted OVA53 cells expressing HA-tagged cyclin D3 were transfected with either *AMBRA1* wild-type (+WT) or patient-type *AMBRA1* mutant (+CS mut). Cell lysates were subjected to IP using an anti-AMBRA1 antibody. The IP products were analyzed by immunoblotting to detect HA (cyclin D3, arrowhead).

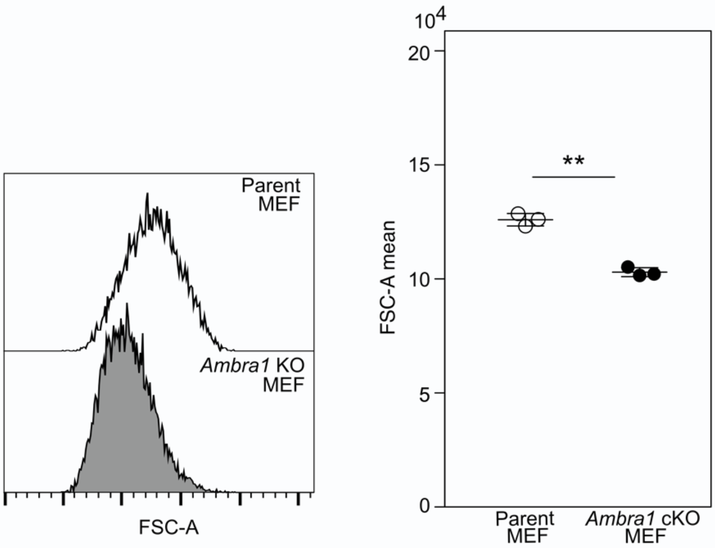

Figure S7 Cell size of *Ambra1* KO MEF is smaller than Parent MEF, related to Figure 5.
Ambra1 deletion was induced by 4-OHT. Cells were subjected to FACS analysis. Cell size was measured by FSC-A in live cells. Data are presented as the mean with SD (**$p < 0.01$ in the Student's t-test).

Akatsuka et al. Figure S8

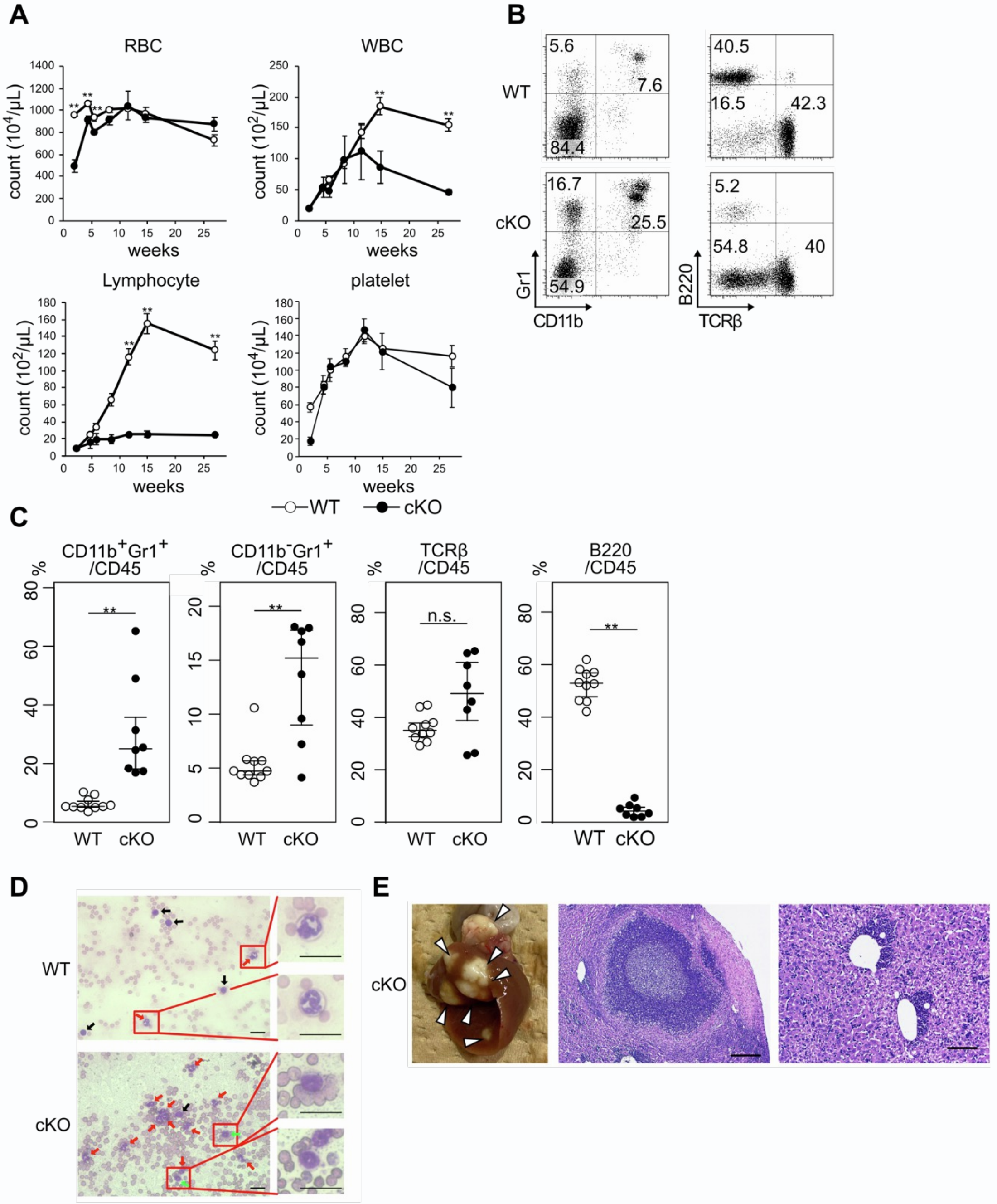

Figure S8 Infiltrating hematopoietic cells were intrinsically affected by *Ambra1* deficiency, related to Figure 5. (A) BM cells of *Ambra1$^{flox/flox}$-Rosa-Cre-ERT2-Tg(+)* and control *Ambra1$^{flox/flox}$-Rosa-Cre-ERT2-Tg(-)* mice were transferred to irradiated normal syngeneic mice. Seven days after the transfer, tamoxifen solution was subcutaneously injected. At the indicated time points, red blood cells (RBC), white blood cells (WBC), lymphocytes, and platelets in the peripheral blood were counted using XT-1800i (Sysmex). Data are presented as means with SEM. (**$p < 0.01$ in the Student's t-test). (B) WBCs were prepared from peripheral blood 15 weeks after BM transfer and examined by FACSverse. Typical FACS profiles of total WBC are shown. (C) The percentage of the cells with the indicated surface phenotypes observed in (B); CD11b$^+$Gr-1$^+$, CD11b$^-$Gr-1$^+$, TCR-β$^+$, and B220$^+$cells among CD45$^+$ cells (open symbols; WT, n = 10, closed symbols; *Ambra1* cKO, n = 8). Data are presented as medians with 25$_{th}$–75$_{th}$ percentiles. (**$p < 0.01$, and n.s.: not significant in the Mann-Whitney U-test). (D) Representative images of Wright-Giemsa staining for peripheral blood from WT and *Ambra1* cKO mice. Red, green, and black arrows indicate mature neutrophils, immature neutrophils, and lymphocytes, respectively. Scale bar, 20 μm. (E) CD11b$^+$Gr-1$^+$ cells originated from transferred BM cells of cKO mice infiltrated (right) and accumulated (middle) in the liver to form large masses (left, arrowheads) scattered throughout the organ. Scale bars, 250 μm (middle) and 100 μm (right).

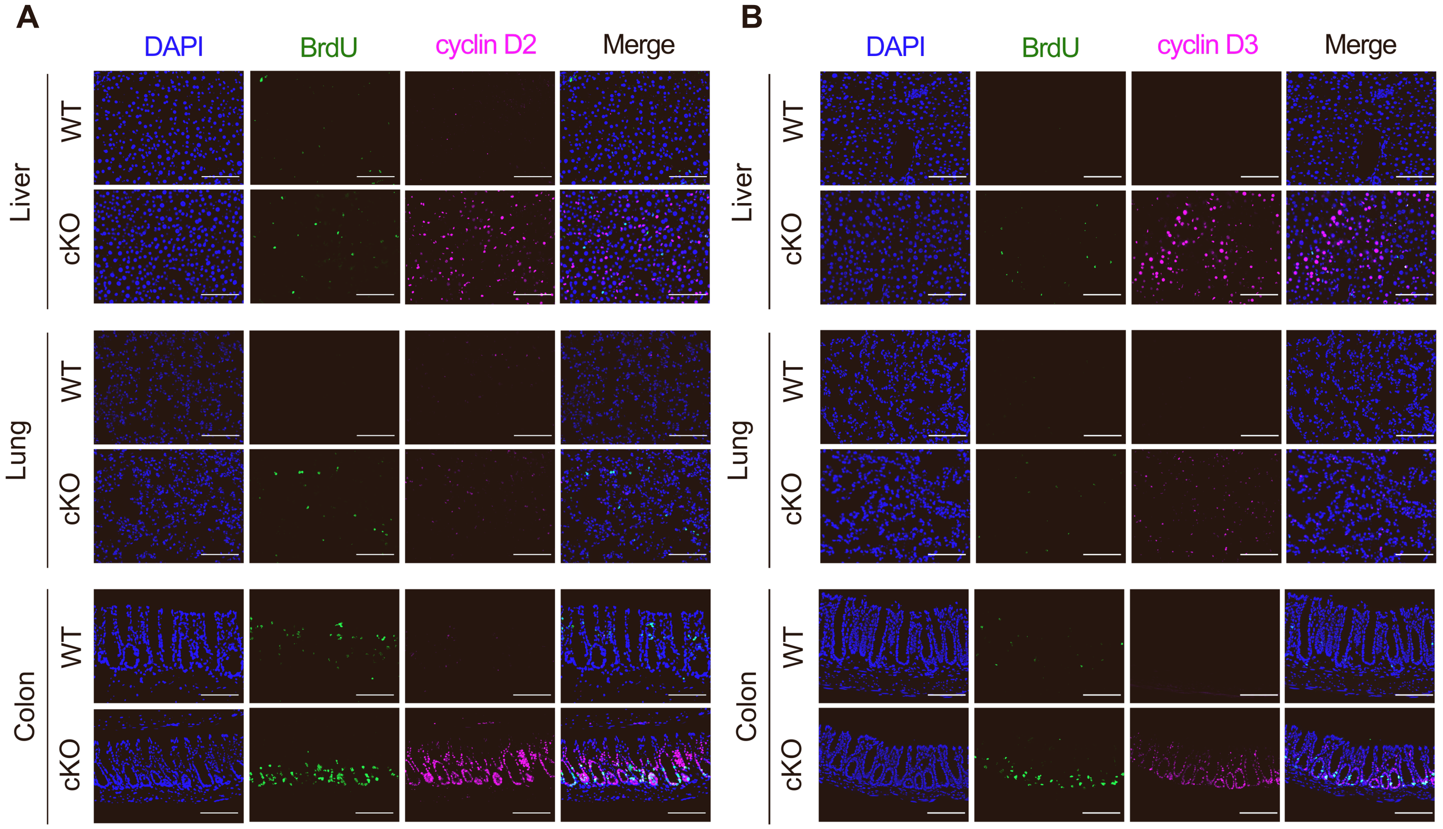

Figure S9 Increase in the number of proliferating cells and cyclin D expression in *Ambra1* cKO mice, related to Figure 6.

(A) Immunohistochemical analysis of BrdU (green) and cyclin D2 (magenta) was performed using fluorescence microscopy. Scale bars, 100 µm.

(B) Immunohistochemical analysis of BrdU (green) and cyclin D3 (magenta) was performed using fluorescence microscopy. Scale bars, 100 µm.

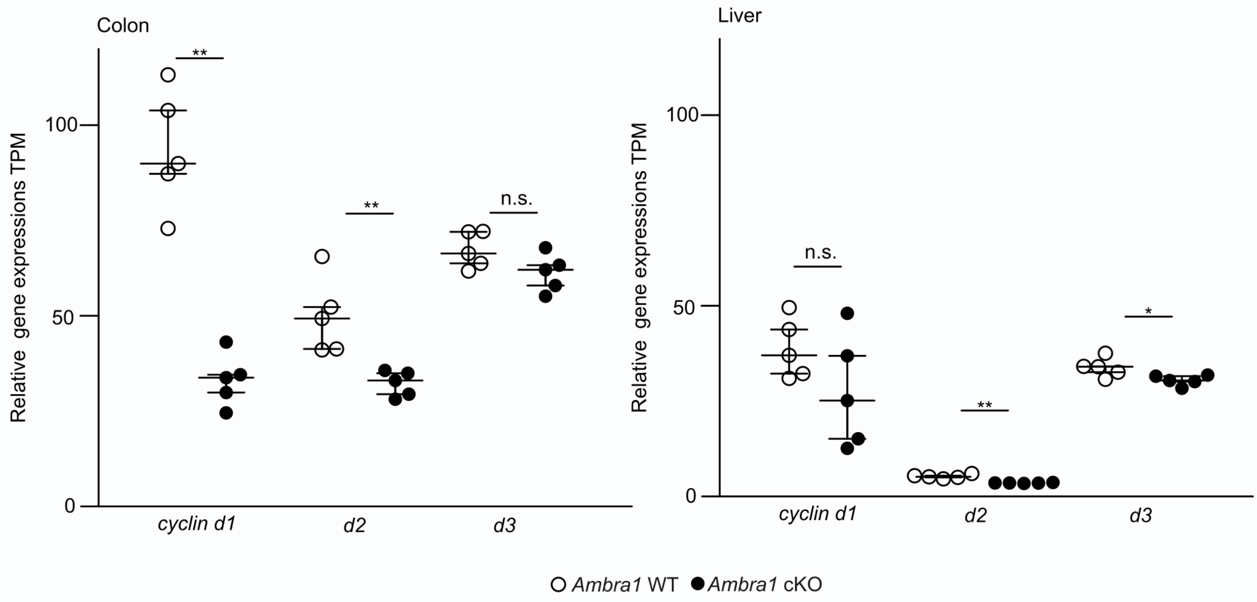

Figure S10 Changes of *cyclin d* expression in *Ambra1* cKO mice, related to Figure 6.

Although the expression of cyclin D proteins increased in *Ambra1* cKO mice, the expression of *cyclin d* transcripts did not increase but often decreased. The expression of *cyclin d1* and *d2* in the colon (left) and *cyclin d2* and *d3* in the liver (right) was significantly decreased in *Ambra1* cKO mice, suggesting a negative feedback mechanism in which the increased expression of cyclin D proteins inhibited its transcription. (n = 5). Data are presented as means with SD (*$p < 0.05$, **$p < 0.01$, and n.s.: not significant, Student's *t*-test).

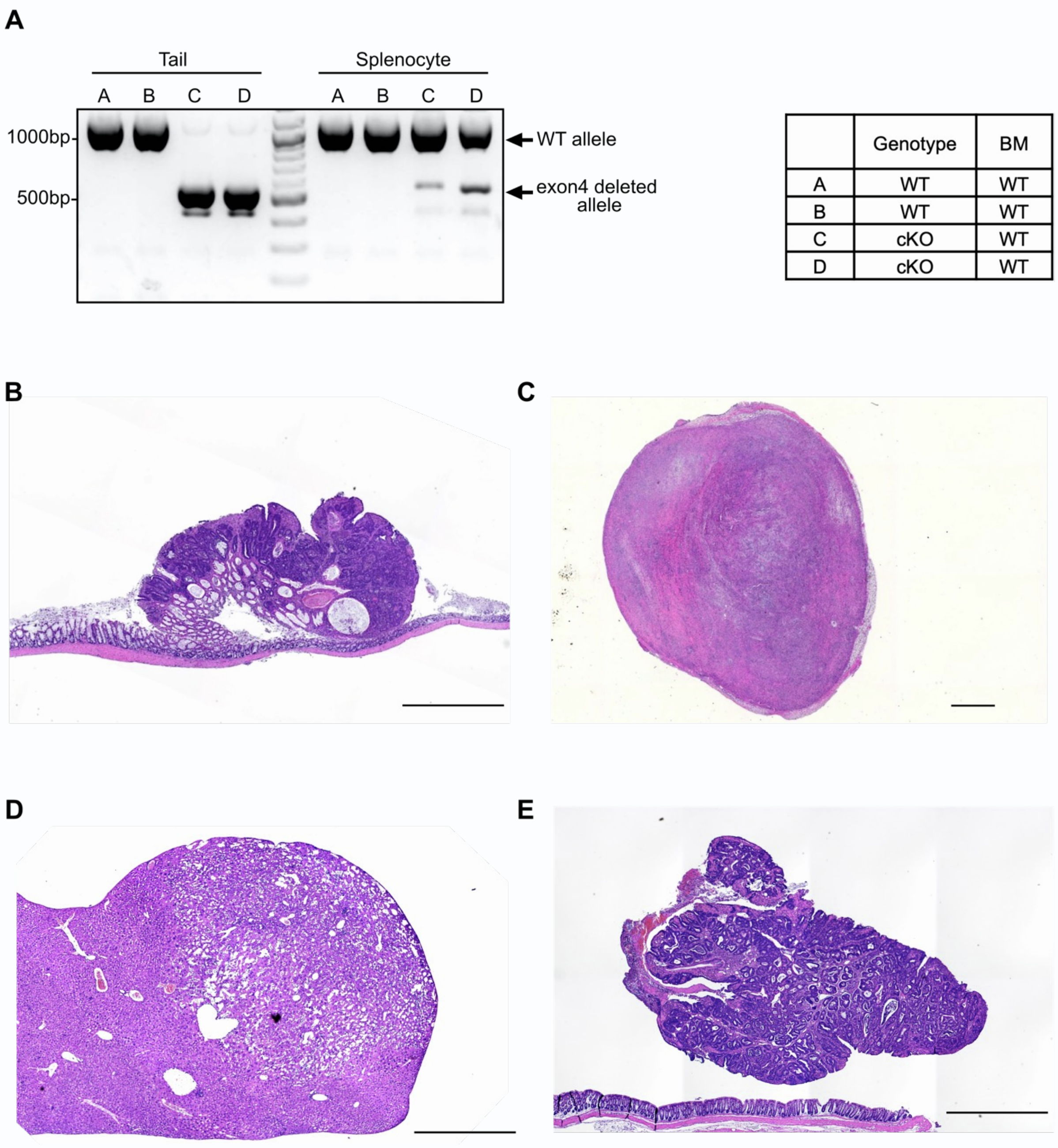

	Genotype	BM
A	WT	WT
B	WT	WT
C	cKO	WT
D	cKO	WT

Figure S11 Increased susceptibility to tumorigenesis of *Ambra1* cKO mice, related to Figure 7.

(A)–(C) Two weeks after tamoxifen administration, *Ambra1*$^{flox/flox}$ mice with or without *Rosa-Cre-ERT2-Tg* were irradiated with 8.5 Gy X-ray and 1×10^6 wild type (WT) bone marrow (BM) cells were transferred. Mice were examined for carcinogenesis after 24 weeks.

(A) Deleted/undeleted *Ambra1* exon 4 was amplified from tail and spleen DNA from the control WT (WT, A and B) and *Ambra1* cKO (cKO, C and D) mice transferred with WT bone marrow cells. PCR analysis confirmed that almost all spleen cells were replaced by transferred *Ambra1*-sufficient cells.

(B) Low-magnification micrograph of the colon cancer shown in Figure. 7G.

(C) A round tumor with a smooth surface in the thoracic space of an *Ambra1* cKO mouse with WT blood cells. Low-magnification micrograph of the section shown in Figure.7H.

(D, E) Azoxymethane (AOM)-induced tumors in the liver (D) and the colon (E) of *Ambra1* cKO mice. Scale bars, 1 mm.

	chromosome	gene_symbol	Substitution	tumor_maf	SIFT_score	SIFT_prediction
Lymphoma (AOM-induced)	chr6	Pdzrn3	p.Ala392Thr; c.1174G>A	0.28947368	0.33	tolerated
	chr6	Gm156	p.Thr61Ile; c.182C>T	0.31428571	0.1	tolerated
	chr10	Mettl24	p.Arg204His; c.611G>A;	0.20338983	0.05	tolerated
	chr11	Ints2,Brip1os	p.Val1009Ile; c.3025G>A	0.1875	0	deleterious
Colon cancerr (AOM-induced)	chr1	Rassf5,Eif2d	p.Gly196Glu; c.587G>A	0.44736842	0	deleterious
	chr1	Enah	p.Ser404Leu; c.1211C>T	0.38095238	0.12	tolerated_low_confidence
	chr3	Iqgap3	p.Arg1409Cys; c.4225C>T	0.2295082	0	deleterious
	chr4	Nol6	p.Ser1038Phe; c.3113C>T	0.33333333	0.02	deleterious
	chr4	Mroh7,Ttc4	p.Ala4Val; c.11C>T	0.23214286	0.09	tolerated
	chr5	Txk	p.Ser423Phe; c.1268C>T	0.32432432	0	deleterious
	chr7	Mboat7,Tmc4	p.Asp396Asn; c.1186G>A	0.33962264	0.06	tolerated
	chr7	Zfp14	p.Gly6Asp; c.17G>A	0.13043478	0.4	tolerated
	chr7	Klk1b24	p.Pro182Ser; c.544C>T	0.37662338	0.99	tolerated
	chr8	Adam3	p.Val21Ile; c.61G>A	0.38888889	0.67	tolerated
	chr8	Tmem184c	p.Ser609Pro; c.1825T>C	0.31707317	0	deleterious_low_confidence
	chr9	Npat	p.Pro1265Leu; c.3794C>T	0.40963855	0.02	deleterious
	chr9	Larp6	p.Asp418Asn; c.1252G>A	0.46376812	0.04	deleterious
	chr9	Tcf12	p.Pro438Ser; c.1312C>T	0.42553191	0.12	tolerated
	chr9	Ctnnb1,Mir7090	p.Gly34Glu; c.101G>A	0.375	0.03	deleterious
	chr10	Tdg,Glt8d2	p.Glu310Lys; c.928G>A;	0.24444444	0.38	tolerated
	chr11	Zmynd15,Cxcl16	p.Gly90Arg; c.268G>A	0.4137931	0	deleterious_low_confidence
	chr11	Fbxo39	p.Arg426Lys; c.1277G>A	0.36363636	0.76	tolerated
	chr12	Grhl1	p.Arg263Gln; c.788G>A	0.25833333	0.06	tolerated
	chr12	Syne2,Mir5101	p.Gly701Asp; c.2102G>A	0.43877551	0.58	tolerated
	chr12	Zdhhc22	p.Ser33Ile; c.98G>T	0.28571429	0.01	deleterious
	chr12	Itgb8	p.Val705Met; c.2113G>A	0.33633634	0.02	deleterious
	chr13	Exoc2	p.Val328Ile; c.982G>A	0.26923077	0.01	deleterious
	chr16	Zbtb21	p.Gly602Ser; c.1804G>A	0.25	0.04	deleterious
	chr18	Mcc	p.Thr887Met; c.2660C>T	0.3220339	0.17	tolerated_low_confidence
	chr19	Pik3ap1	p.Arg186Cys; c.556C>T	0.29268293	0	deleterious
	chr19	Sfr1,Cfap43	p.Val1501Ile; c.4501G>A	0.31707317	1	tolerated
	chr19	Vwa2	p.Ser107Thr; c.319T>A	0.09090909	0.21	tolerated
	chrX	Bcor	p.Val554Ile; c.1660G>A	0.43661972	0.54	tolerated
T-cell lymphoma (AOM-induced)	chr2	Hspa5,Rabepk	p.Pro143Ser; c.427C>T	0.21938776	0	deleterious_low_confidence
	chr4	Svep1	p.Pro1078Leu; c.3233C>T	0.17948718	0.04	deleterious
	chr5	Sgsm1	p.Arg883His; c.2648G>A	0.36893204	0	deleterious
	chr7	Iqgap1	p.Leu328Met; c.982C>A	0.30769231	0.01	deleterious
	chr9	Cspg4	p.Arg2270His; c.6809G>A	0.13333333	0	deleterious
	chr10	Nav3	p.Glu1822Gly; c.5465A>G	0.14847162	0	deleterious
	chr14	Lmo7	p.Glu229Asp; c.687A>C	0.19480519	0.35	tolerated
Thymic lymphoma (spontaneous)	chr2	Plxdc2	p.Ala204Thr; c.610G>A	0.53846154	0	deleterious
	chr2	Strc	p.Arg944Gln; c.2831G>A	0.45333333	0	deleterious
	chr6	Lancl2	p.Arg302Gln; c.905G>A	0.09090909	0.02	deleterious
	chr6	Kras,Gm15706	p.Gly12Asp; c.35G>A;	0.46774194	0	deleterious
	chr7	Cd163l1	p.Cys859Tyr; c.2576G>A	0.42857143	0	deleterious
	chr8	Gtf2e2	p.Pro290Leu; c.869C>T	0.07086614	0	deleterious_low_confidence
	chr8	Fat1	p.Leu918Ile; c.2752C>A	0.41237113	0.05	tolerated
	chr12	Bcl11b	p.Lys828Thr; c.2483A>C	0.57777778	0	deleterious
	chr13	Ryr2	p.Gly1497Arg; c.4489G>A	0.52857143	0.2	tolerated
	chr18	Stk32a	p.Met96Thr; c.287T>C	0.32407407	0	deleterious

Table S4 Somatic mutations in malignant tumor samples of *Ambra1* cKO mouse related to Figure 7.

From the whole-exome sequencing data of spontaneous or AOM-induced malignant tumors arisen in *Ambra1* cKO mice, missense mutations with a variant score of 13.013 or higher, tumor depth of 20 or higher, and tumor alt read of 8 or higher were extracted. Cancer-associated mutations, such as Kras G12D (spontaneous thymic T lymphoma) and Ctnnb1 G34E (AOM-induced colon cancer), were identified.
